# In vivo volumetric imaging of calcium and glutamate activity at synapses with high spatiotemporal resolution

**DOI:** 10.1038/s41467-021-26965-7

**Published:** 2021-11-16

**Authors:** Wei Chen, Ryan G. Natan, Yuhan Yang, Shih-Wei Chou, Qinrong Zhang, Ehud Y. Isacoff, Na Ji

**Affiliations:** 1 Department of Physics, University of California, Berkeley, CA 97420 USA; 2 Department of Molecular and Cell Biology, University of California, Berkeley, CA 94720 USA; 3 Helen Wills Neuroscience Institute, University of California, Berkeley, CA 94720 USA; 4 Molecular Biophysics and Integrated Bioimaging Division, Lawrence Berkeley National Laboratory, Berkeley, CA 94720 USA

**Keywords:** Visual system, Adaptive optics, Multiphoton microscopy

## Abstract

Studying neuronal activity at synapses requires high spatiotemporal resolution. For high spatial resolution in vivo imaging at depth, adaptive optics (AO) is required to correct sample-induced aberrations. To improve temporal resolution, Bessel focus has been combined with two-photon fluorescence microscopy (2PFM) for fast volumetric imaging at subcellular lateral resolution. To achieve both high-spatial and high-temporal resolution at depth, we develop an efficient AO method that corrects the distorted wavefront of Bessel focus at the objective focal plane and recovers diffraction-limited imaging performance. Applying AO Bessel focus scanning 2PFM to volumetric imaging of zebrafish larval and mouse brains down to 500 µm depth, we demonstrate substantial improvements in the sensitivity and resolution of structural and functional measurements of synapses in vivo. This enables volumetric measurements of synaptic calcium and glutamate activity at high accuracy, including the simultaneous recording of glutamate activity of apical and basal dendritic spines in the mouse cortex.

## Abstract (web summary)

Adaptive optics (AO) corrects sample aberrations and allows high spatial resolution at depth in vivo. Here the authors report an AO method for Bessel focus; they apply AO Bessel focus scanning fluorescence microscopy to volumetric imaging and measure synaptic calcium and glutamate activity in vivo.

## Introduction

Imaging biological processes in living organisms ideally require optical microscopy methods with high resolution both spatially and temporally over three dimensions (3D). For example, for in vivo brain imaging, sub-micron spatial resolution is required to resolve synapses, specialized subcellular structures that neurons use to communicate and coordinate activity, while sub-second temporal resolution is required to track neuronal activity. Although it is often desirable and necessary to study synaptic activity in a volume (e.g., across the dendrites of the same neuron), there still lacks methods that can image synapses at high spatiotemporal resolution in 3D at depth.

Among advanced in vivo imaging techniques, two-photon fluorescence microscopy (2PFM)^1^ is the most popular approach for imaging opaque tissues such as the brain, with the minuscule two-photon absorption cross-section restricting fluorescence generation to within the focal volume of a microscope objective. To image a single optical section within a sample, 2PFM scans the excitation focus in two dimensions (2D) and records fluorescence signal at each position, with a diffraction-limited focus providing the brightest fluorescence signal as well as highest spatial resolution. Maintaining high spatial resolution at depth in vivo, however, is only possible via adaptive optics (AO), which measures and corrects for the optical aberrations accumulated on the wavefront of image-forming light while it passes through optically heterogeneous specimen^2,3^. Combining AO with 2PFM, with corrective phase patterns applied to the excitation wavefront at the objective back pupil plane, diffraction-limited performance can be achieved and synapses can be resolved at hundreds of microns below the surface of the brain^4^.

In vivo imaging of the brain also requires high temporal resolution. For functional imaging of a brain volume, sub-second temporal resolution is required to keep pace with the generation and propagation of neuronal activity. A conventional 2PFM achieves 3D imaging by sequentially scanning its excitation focus in three dimensions, which leads to a volumetric imaging rate far below its 2D frame rate^5^. Recently, we and other groups have demonstrated volumetric 2PFM imaging using a Bessel beam as the excitation focus^6–10^. Axially elongated but laterally confined, a Bessel focus enables simultaneous imaging of structures within the volume defined by the 2D scanning area and the axial length of the Bessel focus, converting 2D frame rates into 3D volume rates. Applying Bessel focus scanning 2PFM to the zebrafish larval^11^ and the mouse brains^9,10,12,13^, we demonstrated fast activity imaging of brain volumes while maintaining synapse-resolving lateral resolution.

However, just like the conventional two-photon excitation focus that is formed by fully illuminating the objective back pupil, a Bessel focus, formed by illuminating the pupil with an annular pattern, also experiences sample-induced aberrations that degrade its beam profile after propagating through aberrating media^14–16^. Little is known, however, of how Bessel focus quality is affected by optical aberrations and how we can correct these aberrations in order to recover diffraction-limited performance at depth.

In this study, we theoretically and experimentally characterize how different aberration modes affect the quality of Bessel focus in 2PFM. We develop a highly efficient and effective aberration correction method by manipulating the excitation wavefront at the objective focal plane, instead of the pupil plane as in conventional AO, which enables us to recover diffraction-limited imaging performance. We apply AO Bessel focus scanning 2PFM to volumetric imaging of the zebrafish larval and mouse brains in vivo down to 500 µm depth and demonstrate that aberration correction substantially improves the sensitivity and resolution of both structural and functional measurements of neuronal processes and synapses in vivo. Most importantly, we find that AO is essential for the accurate characterization of synaptic calcium and glutamate activity measured with Bessel focus scanning 2PFM.

## Results

### High-efficiency focal-plane aberration correction for Bessel focus scanning 2PFM

**Fig. 1:**
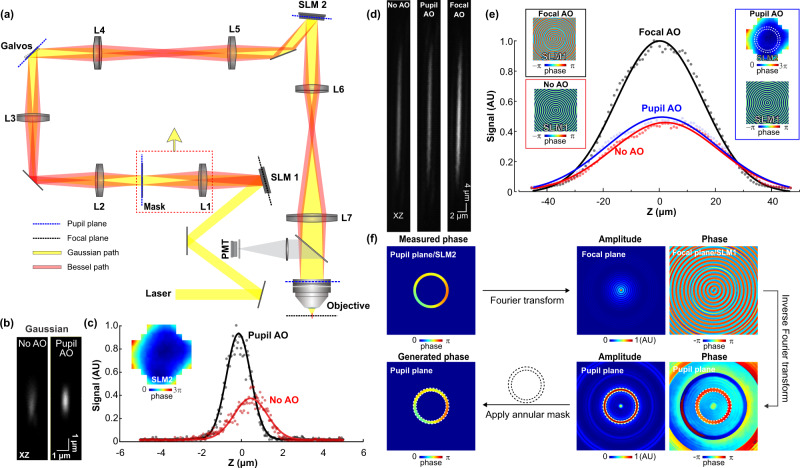
High-efficiency aberration correction for Bessel focus scanning 2PFM. **a** Schematic of AO 2PFM with Gaussian (yellow path) or Bessel (red path) focus scanning. Bessel path has an additional lens (L1) and transmissive annular mask (Mask) placed after the first spatial light modulator (SLM1). Lens pairs (L2–L3, L4–L5, L6–L7) conjugate Mask, X and Y galvanometers (Galvos), another SLM (SLM2), and microscope objective back pupil plane (dashed blue lines). In the Bessel path, SLM1 is conjugated to the objective focal plane (dashed black lines). SLM1: generation and focal-plane aberration correction of Bessel focus; SLM2: indirect wavefront measurement and aberration correction of Gaussian focus. **b** Axial images and **c** signal profiles of a 0.1-µm-diameter fluorescent bead before and after system aberration correction of Gaussian focus. Inset in **c**: pupil-plane corrective wavefront on SLM2. **d** Axial images and **e** signal profiles of a 0.1-µm-diameter fluorescent bead imaged by a Bessel focus without AO (binary phase mask on SLM1 only), with pupil-plane AO (binary phase mask on SLM1, pupil corrective pattern on SLM2), and with focal-plane AO (focal corrective pattern on SLM1 only), respectively. Insets in **e**: phase patterns on SLM1 and SLM2. Dashed circles in Pupil AO inset: footprint of annular illumination on SLM2. **f** Flowchart showing (top) the computation procedure and (bottom) numerical validation of the focal-plane wavefront pattern for aberration-corrected Bessel focus. Top: Pupil-plane corrective pattern within the annular mask is propagated to focal plane via Fourier transform. The resulting phase pattern serves as the aberration-correcting focal-plane phase pattern on SLM1. Bottom: Inverse Fourier transform of the focal-plane phase pattern replicates the annular amplitude and corrective phase patterns on the pupil plane. Concentric dashed circles: annular mask footprints. AU arbitrary unit. Source data are available as a source data file.

An AO Bessel focus scanning two-photon fluorescence microscope (Fig. 1a) was constructed based on a homebuilt AO 2PFM^17^. It had two phase-only liquid-crystal spatial light modulators (SLMs). The first SLM (SLM1) along the 940-nm excitation laser beam path was conjugated to the focal plane of a 25× 1.05 NA water-dipping microscope objective (“focal plane”). A circular binary phase pattern with phase values 0 and π was displayed on SLM1 and diffracted most of the energy of the excitation light into the ±1 diffraction orders (red path, Fig. 1a). A lens (L1) then focused the laser to an annular transmission mask, which spatially filtered the excitation light and was optically conjugated to two galvanometers, the second SLM (SLM2), as well as the back pupil plane of the objective (“pupil plane”), to generate a 0.4-NA Bessel focus with an axial full-width at half-maximum (FWHM) of 43 µm for two-photon fluorescence excitation.

To switch to the more conventional “Gaussian” excitation focus, which was generated when the laser illumination at the pupil plane had a Gaussian intensity profile, a flat phase was applied to SLM1, with the annular mask and L1 moved out of the beam path (Supplementary Fig. 1). With the laser following the Gaussian beam path (yellow path, Fig. 1a), we measured the wavefront aberrations experienced by the excitation light in the microscope system itself via a pupil-segmentation-based AO method (Supplementary Fig. 2a, Methods)^17^. Displaying the corrective wavefront (inset, Fig. 1c) on SLM2 to cancel out the optical system aberration, we increased the fluorescence signal of a 0.1-µm-diameter fluorescent bead by 3× and achieved diffraction-limited resolution (axial FWHM = 1.08 µm for a 1.05-NA Gaussian focus of 940 nm excitation light) after AO correction (Fig. 1b, c). When we used Bessel illumination, however, the corrective pattern on SLM2/pupil plane only increased the bead signal by ~10% (Fig. 1d, middle panel, “Pupil AO”; Fig. 1e). This was not because the Bessel focus was more resistant to aberrations or more sensitive to residual phase error (Supplementary Note and Supplementary Fig. 3). Instead, phase aberrations that excitation light experienced away from the pupil plane caused both phase and amplitude distortions at the pupil plane. A phase-only correction in the pupil plane was unable to correct amplitude distortion, which caused larger signal degradation for Bessel foci (Supplementary Note and Supplementary Fig. 4a–d).

To achieve high-efficiency aberration correction for Bessel foci, we corrected for both phase and amplitude distortions by using SLM1, which was fully illuminated by our excitation laser and conjugated to the focal plane, for aberration correction (“Focal AO”). To account for the effect of aberrations, instead of the circular 0–π binary phase pattern, we determined the optimal phase pattern on SLM1 computationally (upper panels, Fig. 1f and Supplementary Fig. 2b). We first fit the SLM2/pupil-plane corrective pattern with the first 55 Zernike modes and removed tip, tilt, and defocus from the fitted pattern. We then applied the annular amplitude mask onto the corrective pattern and Fourier-transformed the phase pattern within the annulus to obtain the amplitude and phase of the complex electric field distribution on the focal plane. Finally, we applied a flat phase pattern to SLM2, which now simply acted as a mirror, and displayed the phase-only map as calculated above on SLM1 for both the generation and aberration correction of the Bessel focus. This phase-only correction in the focal plane, when propagating to the pupil plane, corrected for both phase and amplitude distortions of the excitation light, and therefore led to much higher recovery of excitation intensity than pupil AO (Supplementary Fig. 4e, f).

Using the microscope system aberration as an example, the focal AO/SLM1 pattern was calculated from the corrective phase pattern at the pupil plane (upper panels, Fig. 1f and Supplementary Fig. 2b). The Bessel illumination light generated by the SLM1 pattern, when propagated to the SLM2/pupil plane (calculated via an inverse Fourier transform) had the desired corrective phase for aberration correction (lower panels, Fig. 1f and Supplementary Fig. 2c). Experimentally, focal AO improved the signal of a 0.1-µm-diameter fluorescent bead excited by the Bessel focus by 2.2× (Fig. 1d,e), an improvement much larger than pupil AO correction (1.1×). From here on, all “No AO” images were taken with the microscope system aberration corrected, so that the image quality improvements after AO correction arose from correcting sample-induced aberrations.

### Aberration modes differentially degrade signal and point spread function profiles of Gaussian and Bessel foci

**Fig. 2:**
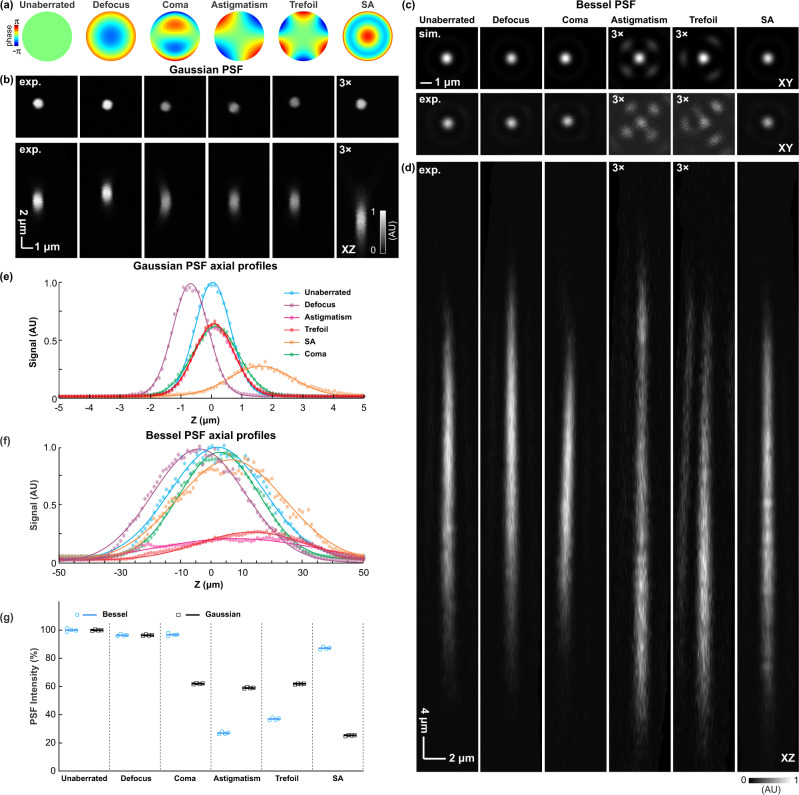
Aberration modes differentially degrade signal and point spread function (PSF) profiles of Gaussian and Bessel foci. **a** Zernike modes representing defocus, coma, astigmatism, trefoil, and spherical aberrations (SA), respectively, are introduced to the pupil plane (SLM2). **b** Lateral (XY) and axial (XZ) PSFs of the Gaussian focus measured using a 0.1-µm-diameter fluorescent bead. **c** Lateral PSFs of the Bessel focus (upper row) simulated and (lower row) measured using a 0.1-µm-diameter fluorescent bead. **d** Axial PSF of the Bessel foci measured using a 0.1-µm-diameter fluorescent bead. **e**, **f** Axial profiles of the Gaussian and Bessel PSFs, respectively. **g** Peak signals of Gaussian and Bessel PSFs under different aberration modes. Normalized to the unaberrated PSFs. *n* = 4 measurements for each aberration mode were acquired from the same bead. Data are presented as dot plots with lines representing mean values. AU arbitrary unit. Source data are available as a source data file.

As shown in Fig. 1, Bessel focus, despite of being often cited for its capability of “self-healing”, is indeed susceptible to optical aberrations. We further investigated how different aberration modes impact Bessel PSFs in 2PFM. We applied wavefront distortions of selected low-order Zernike aberration modes with coefficients of 1 wave (i.e., Zernike modes corresponding to defocus, coma, astigmatism, trefoil, and spherical aberration, ${Z}_{2}^{0}$, ${Z}_{3}^{1}$, ${Z}_{2}^{2}$, ${Z}_{3}^{3}$, ${Z}_{4}^{0}$, Fig. 2a) to SLM2/pupil plane, and measured the two-photon fluorescence signal as well as lateral and axial profiles of 0.1-µm-diameter beads excited by the 1.05-NA Gaussian and 0.4-NA Bessel foci, respectively (Fig. 2).

For both Gaussian and Bessel foci, the small defocus shifted them axially and did not impact signal or resolution substantially, as expected (Fig. 2b–f). For the Gaussian focus, all other aberration modes led to substantial degradation in its fluorescence signal and enlargement of its axial PSF (Fig. 2b, e, g). However, for Bessel PSFs, coma and spherical aberrations minimally impacted their peak signals, whereas astigmatism and trefoil reduced their signals more than they did for Gaussian PSFs (Fig. 2d, f, g). The experimentally measured Bessel PSFs of astigmatism and trefoil indicated that more excitation energy was distributed into side lobes (Fig. 2c, lower row), with patterns matching those calculated using a numerical model that took noncircularly symmetric aberrations into consideration (Fig. 2c, upper row; Supplementary Note)^18^. The robustness of Bessel focal quality against circularly symmetric aberrations such as spherical aberration can be explained by its annular illumination pattern at the pupil plane, where circularly symmetric aberrations caused minimal phase errors between light rays. The same argument does not explain why coma, an aberration mode without circular symmetry, did not degrade Bessel PSF. We developed an analytical solution for the focal electric field of ideal Bessel beams aberrated by Zernike polynomials ${Z}_{n}^{m}$, and found that ideal Bessel beams are only sensitive to aberration modes with the azimuthal index *m* larger than 1 (Supplementary Note). Given that the applied coma aberration corresponded to ${Z}_{3}^{1}$ in the Zernike polynomials, this explained why it minimally reduced Bessel PSF intensity. Because biological tissues introduce complex aberration patterns, below we applied the focal plane AO method described in the previous section to characterizing how AO correction for Bessel focus improved in vivo volumetric morphological and functional imaging of the mouse and zebrafish larval brains, and compared it with AO correction for Gaussian focus through the same tissues.

### AO improves volumetric imaging of dendritic and synaptic morphology by Bessel focus scanning in the mouse primary visual cortex in vivo

We first tested how AO correction of sample-induced aberration improves in vivo volumetric morphological imaging of neurons in the mouse brain. We installed a cranial window made of a No. 1.5 coverslip over the primary visual cortex (V1) of a *Thy1*-GFP line M mouse, and imaged the pyramidal neurons expressing the fluorescent protein GFP down to a depth of 500 µm.

We first imaged dendritic structures in the superficial cortex (Supplementary Fig. 5), where aberration mainly arose from the refraction index mismatch between the cranial window and the immersion water for which the 1.05-NA objective was designed. We measured the cranial-window-induced aberration of the Gaussian focus using a 2-µm-diameter fluorescent bead embedded between the window and the brain. Correcting the aberration increased the fluorescence signal of the bead imaged with the Gaussian focus by 2.2-fold and achieved diffraction-limited resolution (axial FWHM of the bead: 2.6 µm, Supplementary Fig. 5a). From the pupil-plane corrective wavefront for the Gaussian focus, we then calculated the focal-plane wavefront pattern for aberration-corrected Bessel focus generation (Supplementary Fig. 5b) and acquired images of the same volume using either Gaussian or Bessel focus scanning without and with aberration correction (XY: 128 × 128 µm^2^; 40–100 µm below pia, Supplementary Fig. 5c–h). Larger (~2×) average power was used with Bessel focus scanning, because higher fraction of power was distributed to the side rings for the Bessel focus than for the Gaussian focus (Supplementary Fig. 6). For both Gaussian and Bessel foci, correcting the cranial window aberration resulted in higher resolving power and brighter fluorescence signal for dendrites and spines located at shallow cortex depths. Because the main aberrations introduced by the cranial window were Zernike modes corresponding to primary spherical (for a cranial window perpendicular to the optical axis) or coma aberration (for a tilted window)^19^, Bessel focus was less affected by the cranial window aberrations and the maximal signal improvement by AO in the Bessel images (2.1×) was smaller than the improvement in the Gaussian images (2.5×) (Supplementary Fig. 5g).

**Fig. 3:**
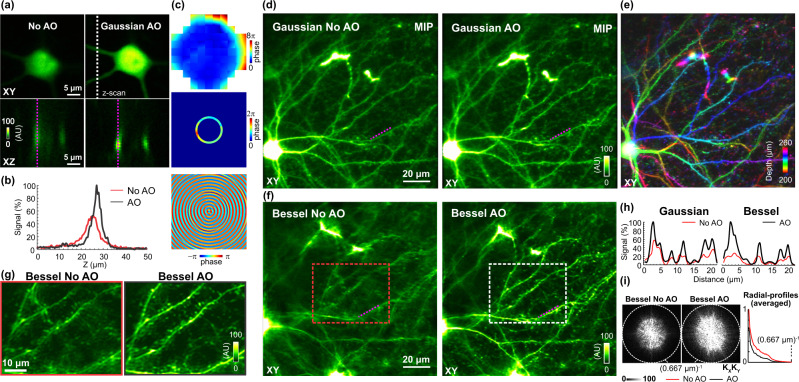
AO improves volumetric imaging of dendritic and synaptic morphology of mouse cortex in vivo. Primary visual cortex of a *Thy1*-GFP mouse was imaged through a cranial window in vivo. **a** Lateral images of a soma (*Z* = 230 µm) and axial images (along the dashed white line) of nearby dendrites measured without and with AO correction of Gaussian focus. Aberration measurement was performed using the soma. **b** Signal profiles of dendrites along the dashed purple lines in **a**. **c** Wavefront on pupil plane/SLM2 for aberration correction of Gaussian focus, after transmitted through the annular mask, and the computed focal-plane pattern on SLM1 for aberration-corrected Bessel focus. **d** Mean intensity projections (MIP) of Gaussian imaging stacks from *Z* = 200 µm to *Z* = 260 µm without and with AO. **e** Gaussian imaging stack with AO color-coded by depth. **f** Bessel images of the same volume as in **d**, obtained without and with AO. Lateral pixel size: 0.5 µm. **g** Zoomed-in images of the volume within dashed boxes in **f**. Lateral pixel size: 0.2 µm. **h** Signal profiles of dendritic spines along the dashed purple lines in **d** and **f**. **i** Spectral power in the spatial frequency space (K_X_K_Y_) for Bessel images in **g** (0.2 µm pixel size) and their radially averaged profiles. Dashed circle: diffraction limit. Post-objective powers: 36 mW for Gaussian and 78 mW for Bessel. Wavelength: 940 nm. AU arbitrary unit. Source data are available as a source data file.

At larger depths, however, brain tissue started to introduce additional aberrations. We measured the window and tissue aberrations using the cell body of a neuron at 230 µm below the brain surface. Correcting the aberration of the Gaussian focus increased both fluorescence signal and spatial resolution (Fig. 3a), leading to brighter, more axially confined dendritic morphology (Fig. 3b). Similar improvements in image quality were observed in a 3D Gaussian image stack (XY: 128 × 128 µm^2^; 1 µm *Z* step size; 200 µm to 260 µm below pia; mean intensity projections, Fig. 3d, e) of dendrites and spines in the surrounding volume. From the pupil-plane corrective wavefront for Gaussian focus, which for this brain included substantial trefoil contribution, we derived the focal-plane wavefront pattern to generate an aberration-free Bessel focus in the brain (Fig. 3c). We scanned the Bessel focus in 2D to generate a projected image of dendrites and dendritic spines of the same volume. Compared with AO correction for the Gaussian images, more pronounced improvements in resolution and signal were observed in the Bessel images (Fig. 3f, g). Whereas AO correction increased the signal of dendritic spines in the Gaussian images by up to 3×, correcting aberration for the Bessel focus led to up to 4× increase in signal of the same spines (Fig. 3h). This is consistent with the data in Fig. 2, where trefoil was found to more severely degrade Bessel than Gaussian foci. The improvement of spatial resolution led to the detection of more dendritic spines (Fig. 3f, g) and can also be appreciated in the Fourier domain (Fig. 3i), with the aberration-corrected image having larger amplitudes across all frequency components but especially for the high spatial frequency components closer to the diffraction limit (dashed white circles, Fig. 3i). Using the same approach, we also measured and corrected the sample-induced aberration for a neuron in layer 5 of mouse V1 (depth of soma: 500 µm) (Supplementary Fig. 7). We observed similar improvements in the Bessel image of dendrites and dendritic spines within a volume spanning 480–540 µm depth. Using a parameter-free image resolution estimation method based on decorrelation analysis^20^, we further confirmed that near-diffraction-limited resolution was achieved for Bessel images after AO correction (Supplementary Fig. 8). In addition to synaptic imaging in mouse, we also tested our approach in volumetric imaging of the zebrafish larval hindbrain. Using an exogenously introduced fluorescence bead for pupil-segmentation AO correction and our focal AO method for correcting sample aberrations, we observed substantial improvements in resolution and brightness of volumetric images of motor neurons and their neuronal processes (Supplementary Note and Supplementary Fig. 9). Together, these results validated the performance of our focal-plane aberration correction method in vivo and indicated that AO was essential for the volumetric morphological measurement of neuronal processes and synapses at depth in different model organisms.

### AO correction enables accurate characterization of orientation tuning properties of dendritic spines in the awake mouse V1 in vivo with calcium imaging

**Fig. 4:**
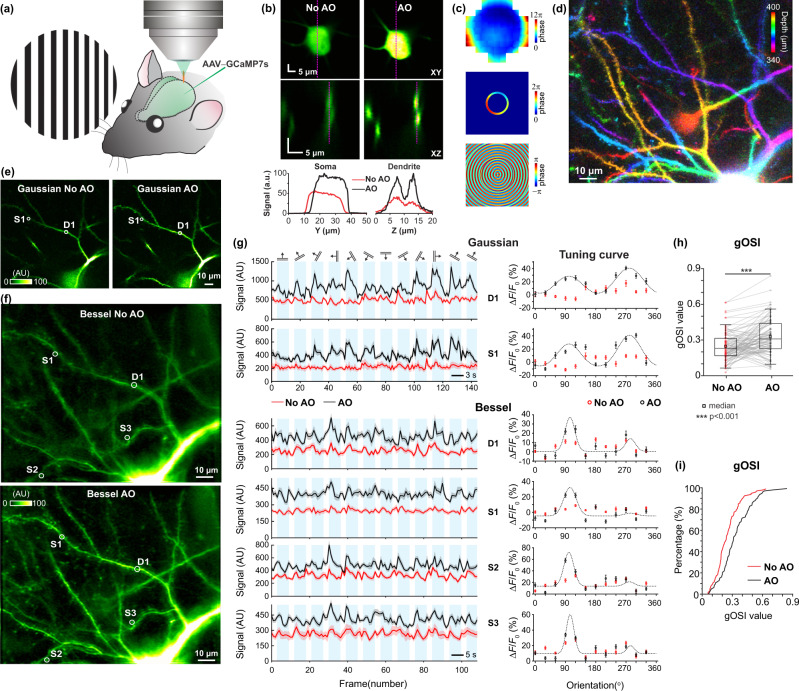
AO enables accurate characterization of orientation-tuning properties of volumes of dendritic spines in awake mouse V1 with in vivo calcium imaging. **a** Mouse V1 sparsely expressing the genetically encoded calcium indicator GCaMP7s was imaged through a cranial window in vivo, while the mouse was presented with 12 drifting-grating stimuli, each repeated for 10 trials. **b** (upper) Lateral images of a soma (*Z* = 390 µm) and (middle) axial images of its nearby dendrites measured without and with AO correction of Gaussian focus; (lower) Signal profiles along the dashed purple lines. Aberration measurement was performed using the soma. **c** Wavefront on pupil plane/SLM2 for aberration correction of Gaussian focus, after transmitted through the annular mask, and the computed focal-plane pattern on SLM1 for aberration-corrected Bessel focus. **d** Gaussian imaging stack from *Z* = 340 µm to *Z* = 400 µm color-coded by depth. **e** A single optical section (*Z* = 350 µm) image with Gaussian focus, measured without and with AO. **f** Bessel images of the same volume as in **d**, obtained without and with AO. **g** Trial-averaged calcium transients (*n* = 20 trials) evoked by 12 drifting-grating stimuli and corresponding tuning curves for one dendrite (D1) and three spines (S1–3), measured with Gaussian and Bessel foci, without and with AO, respectively. Only tuning curves that passed the statistical criteria for orientation selectivity were fitted (see “Methods”). Shadows and error bars: SEM. **h** Global orientation selectivity indices (gOSIs) for *n* = 91 active spines from the volume imaged by Bessel focus scanning without and with AO. Box-and-whisker plots: center line, median; box upper and lower limits, 25th and 75th percentile; whiskers, SD. Two-sided paired *t*-test, ***p < 0.001. **i** Cumulative distributions of gOSI. Kolmogorov–Smirnov test, *p* < 0.001. Post-objective powers: 108 mW for Gaussian and 97 mW for Bessel. Wavelength: 940 nm. AU arbitrary unit. Source data are available as a source data file.

An important application of in vivo optical microscopy in neuroscience is investigating neuronal activity in synaptic terminals and dendritic compartments associated with sensory stimuli or motor outputs^21,22^. Here, we evaluated how AO correction of sample-induced aberration improved volumetric measurements of calcium activity in dendrites and dendritic spines in mouse V1, where neurons are known to selectively respond to drifting gratings of specific orientations (Fig. 4a).

In a mouse with V1 neurons sparsely expressing GCaMP7s, we measured the sample-induced aberrations of the Gaussian focus on a cell body at 390 µm depth. After aberration correction, we observed increase in the basal fluorescence signals of both the cell body and its nearby dendrites (by averaging the frames without elevated somatic calcium transients, Fig. 4b). From the pupil-plane corrective pattern, we derived the focal-plane wavefront pattern for aberration-corrected Bessel focus (Fig. 4c). We then measured the drifting-grating-induced calcium signals from this neuron using Gaussian and Bessel focus scanning, respectively (oriented drifting gratings in 12 directions, presented in pseudorandom sequence, 10 trials for each stimulus). Scanning the Bessel focus in 2D enabled us to measure activity from a volume of dendrites and dendritic spines ranging from 340 to 400 µm below pia (Fig. 4d, f) simultaneously, whereas scanning the Gaussian focus only allowed us to image a few dendritic spines within the much more restricted axial excitation profile (Fig. 4e and Supplementary Movies 1 and 2). AO correction substantially improved the signal and contrast of both Gaussian and Bessel basal fluorescence images (Fig. 4e, f). For the aberration-corrected Bessel image, we observed 91 spines within a 128 × 105 × 60 µm^3^ volume, 54 of which were unresolvable in the Bessel image without AO correction. Similar signal and resolution improvements were also observed in the spontaneous activity of dendritic spines that expressed GCaMP6s in mouse V1 (Supplementary Fig. 10, Supplementary Note and Supplementary Movies 3 and 4).

Importantly and consistent with what we observed previously in the Gaussian focus scanning modality^23–26^, correcting optical aberration also led to more accurate characterization of the functional properties of neurons. For both Gaussian and Bessel images, as indicated by the example trial-averaged fluorescence traces for each grating stimulus (Fig. 4g), more calcium transients were detected for both dendrites and dendritic spines. Among all spines (*N* = 91) identified in AO Bessel image, 58% (53 spines) exhibited visually evoked activity (i.e., ∆*F/F*_*0*_ > 20%), whereas only 21% of spines (19 spines) exhibited calcium transients in the experiment without AO correction. As indicated by the tuning curves of the example dendrites and spines (Fig. 4g), the increased ability towards detecting calcium transients after aberration correction also led to the discovery of their orientation selectivity. In contrast, their tuning curves measured without AO failed to pass the statistical test used to define orientation-selective responses (*p* < 0.05, one-way ANOVA across 12 directions, Methods). We calculated the global orientation-selectivity index (gOSI, Methods) for each individual spine and compared the gOSI distributions measured with and without AO. We found that aberration correction significantly increased the measured orientation selectivity of these spines (without AO, median = 0.23; with AO, median = 0.30; Fig. 4h, paired *t*-test, *p* < 0.001; Fig. 4i, Kolmogorov–Smirnov test, *p* < 0.001; *N* = 91 spines), suggesting that AO is essential for the accurate characterization of functional responses of synapses at depth.

### AO correction enables simultaneous volumetric imaging of glutamate release at both apical and basal dendritic spines with high sensitivity

As the most abundant excitatory neurotransmitter in the vertebrate nervous system, glutamate release directly reflects excitatory neuronal activity at synapses and can be probed by genetically encoded glutamate sensors such as the intensity-based glutamate-sensing fluorescence reporter (iGluSnFR)^27^. With the glutamate sensor tethered to and labeling cell membrane (in contrast to the calcium sensors, which are typically expressed in the cytosol), its fluorescence signal is more sensitive towards the presence of aberrations^28^, because signal from smaller fluorescence structures is more degraded by a distorted excitation focus^23^. Furthermore, because glutamate sensors tend to have faster temporal dynamics and lower brightness than calcium sensors, to capture glutamate dynamics in 3D, it is even more essential to have a fast volumetric imaging method with high sensitivity. With AO Bessel focus scanning 2PFM, here we demonstrated simultaneous volumetric imaging of glutamate activity from apical and basal dendritic spines of a L2/3 neuron.

**Fig. 5:**
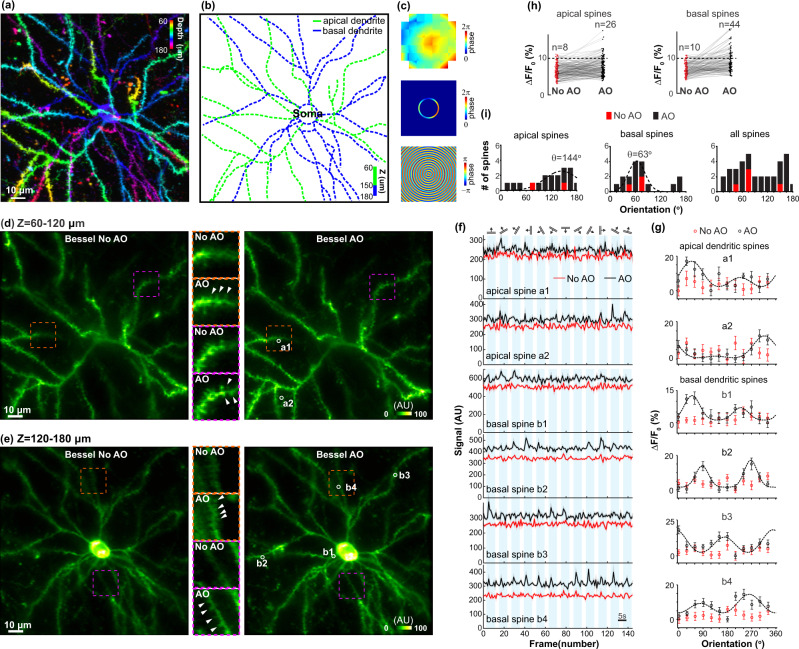
Volumetric imaging of visually evoked glutamate release at apical and basal dendritic spines of a mouse V1 neuron in vivo with AO-corrected Bessel focus. **a** Mean intensity projection of the Gaussian imaging stack (128 × 128 × 120 µm^3^) covering both apical and basal dendritic branches (color-coded by depth). **b** Apical (green curve) and basal (blue curve) dendritic branches identified from the Gaussian stack. **c** Wavefront on pupil plane/SLM2 for aberration correction of Gaussian focus, after transmitted through the annular mask, and the computed focal-plane pattern on SLM1 for aberration-corrected Bessel focus. **d**, **e** Simultaneously imaged apical and basal dendritic branches of the same volume as in **a** with Bessel focus before and after AO correction. Insets: zoomed-in views of the structures in dashed boxes. White arrows: spines only resolvable after AO. **f** Trial-averaged (*n* = 20 trials) glutamate transients of representative apical and basal dendritic spines (white circles in **d** and **e**) evoked by 12 drifting-grating stimuli before and after AO correction and **g** their corresponding tuning curves averaged across *n* = 20 trials from a mouse. Shadows (**f**) and error bars (**g**): SEM. **h** Glutamate transient amplitudes (∆*F*/*F*_0_) for 182 apical and 143 basal dendritic spines before and after AO. *n*: number of spines with ∆*F*/*F*_0_ > 10%. **i** Preferred orientation distributions of apical, basal, and all dendritic spines measured without (red) and with (black) AO. Dashed curves: Gaussian fits to identify dominant orientations. Post-objective powers: 92 mW for Gaussian and 116 mW for Bessel. Wavelength: 940 nm. AU arbitrary unit. Source data are available as a source data file.

Using Gaussian focus scanning, we took a 3D image stack of a mouse V1 neuron expressing the glutamate sensor iGluSnFR.A184S (Fig. 5a) and identified the apical and basal dendritic branches of the neuron based on their morphology (Fig. 5b). Sample-induced aberrations were then measured using signal from its cell body and the corrective wavefront in the focal plane calculated for Bessel focus (Fig. 5c). Using the piezoelectric holder of the objective, we alternately scanned the Bessel focus rapidly between two depths dominated by apical and basal dendrites, respectively. As a result, we were able to monitor the volumetric glutamate dynamics of both apical and basal dendrites simultaneously over a 128 × 128 × 120 µm^3^ volume at 1.6 volumes per second, while presenting drifting-grating stimuli (drifting in 12 directions, presented in pseudorandom sequence, 20 trials for each stimulus) to the mouse (Supplementary Movies 5 and 6).

Comparing the Bessel images taken without and with AO correction, we found that many apical and basal dendritic spines were only identifiable after AO correction (e.g., arrowheads in the zoomed-in views in Fig. 5d, e). Most importantly, by increasing the signal and resolution of dendritic spine imaging, AO correction substantially improved the sensitivity of glutamate detection, with glutamate transients often only visible after brain-induced aberrations were removed (Fig. 5f). As a result, the orientation-tuning properties of these spines can only be accurately measured with AO Bessel focus scanning 2PFM (Fig. 5g, the orientation selectivity of the six example spines only manifested itself in their tuning curves after AO correction). Defining spines with visually evoked responses as those with $\triangle F/{F}_{0} > 10 \%$, we found that 26 out of 182 apical spines and 44 out of 143 basal spines showed responses to drifting gratings in the data obtained with aberration-corrected Bessel focus, whereas only 8 apical and 10 basal spines showed visually evoked responses without AO correction (Fig. 5h). Among the spines with visually evoked activity, 18 apical and 17 basal spines showed orientation selectivity with AO, whereas only 2 apical spines and 3 basal spines passed the orientation selectivity test (*p* < 0.05, one-way ANOVA across 12 directions) without AO.

With the focal AO method enabling accurate measurements of glutamate dynamics at high sensitivity, we were able to compare the preferred orientation distributions of spines on apical and basal dendrites (Fig. 5i). Interestingly, we found an 81° shift in the dominant orientations of apical versus basal dendritic spines (144° versus 63°, as determined by fitting preferred orientation histograms with a Gaussian function; dashed curves, Fig. 5i). The apical dendritic spines also possessed a broader distribution of preferred orientations than basal dendritic spines (FWHMs of the Gaussian peaks: apical, 92°; basal, 46°). The broader distribution in apical spines may arise from the more diverse long-range inputs in cortical L1. Our observation of the almost orthogonal shift in the preferred orientation distributions between inputs onto apical and basal dendrites is consistent with a previous study that showed a shift in the preferred orientation of soma after basal dendrites ablations^29^.

## Discussion

An ideal Bessel beam is non-diffractive and maintains its lateral confinement without spreading out during propagation^30^. Bessel-like beams propagating over large (but finite) distances without substantial diffractive spreading can be experimentally realized^6,30^. Their unique properties have been extensively exploited for the manipulation of matter^31^, material fabrications^32^, and, as detailed above, microscopy. Previous studies reported that compared to Gaussian beams, Bessel beams are more resistant to wavefront perturbation with self-reconstructing capability^33–36^. However, other studies showed that Bessel beam can be degraded by aberrations^15,37–39^ and the presence of self-healing depends on the nature of the disturbance, the location of the measurement, and the metrics chosen to characterize beam quality^14,15^. In this study, we systematically investigated how different aberration modes impact a Bessel beam used for fluorescence excitation of a 2PFM. Experimental measurements, numerical simulations, and theoretical analysis all led to the same conclusions that Bessel beams are more affected by aberration modes such as astigmatism and trefoils than Gaussian beams, but are resistant to aberrations modes corresponding to Zernike polynomial ${Z}_{n}^{m}$’s with ${{{{{\boldsymbol{|}}}}}}{m|}\le 1$ (e.g., spherical aberration and coma). The later discovery is applicable to Bessel beams in general, and provides a theoretical explanation of why in certain experimental systems, where aberrations are dominated by ${Z}_{n}^{m}$’s with ${{{{{\boldsymbol{|}}}}}}{m|}\le 1$, Bessel beams can maintain beam quality with propagation but Gaussian beam cannot.

Since aberrations introduced by biological samples can encompass a variety of aberration modes, to achieve diffraction-limited volumetric imaging with Bessel focus scanning 2PFM, we need to measure and correct the distortion on the excitation wavefront of the Bessel focus. Conventional AO strategies apply corrective wavefront at a plane conjugate to the objective back pupil. We found that such a pupil AO approach, although worked well for Gaussian foci, did not lead to effective aberration correction for Bessel foci. This is because aberrations occurring away from the pupil plane distort both the phase and the amplitude of the pupil electric field. Correcting the phase distortion with a phase-only wavefront corrector at the pupil plane alone leaves the amplitude distortion uncorrected, which reduces the Bessel focal intensity more severely than it does to Gaussian foci. Instead, we developed an algorithm that calculated the wavefront on a plane conjugated to the objective focal plane, which after Fourier transformation corrects both the phase and amplitude distortions at the pupil plane and generates an aberration-corrected Bessel focus within the sample. It is noteworthy that the proposed focal AO method is distinct from the “conjugate AO” methods, which place one or more wavefront correction elements (e.g., deformable mirrors) in planes conjugate to dominant aberration-inducing layers (typically distinct from the pupil or focal planes) to increase the isoplanatic patch of the corrective wavefront^40^.

This focal-plane aberration correction approach can be generally applied to other imaging methods to remove aberrations from image-forming light with a small pupil footprint. Indeed, a similar focal-plane correction approach was previously developed to generate aberration-corrected lattice excitation patterns in a light-sheet microscope^41^. Although here we used an indirect pupil-segmentation-based wavefront-sensing method to obtain the pupil-plane corrective pattern, our focal-plane correction method can also be combined with other aberration measurement methods, including those using direct or modal wavefront sensing^2,42^. Once the pupil aberration is measured, the wavefront pattern on the focal plane is calculated and displayed on SLM1 to generate an aberration-free Bessel focus. (We note that although either deformable mirrors or liquid-crystal SLMs could be used for pupil AO methods, because the corrective pattern at the focal plane is discontinuous with high spatial frequency, deformable mirrors that are accessible to microscopists are not realistic options for focal AO correction of Bessel focus.)

Applying AO Bessel focus scanning 2PFM to in vivo brain imaging of neuronal processes and synapses, we were able to fully recover diffraction-limited resolution, with the aberration-corrected volumetric images possessing higher signal, contrast, and resolution. These improvements arose from better lateral confinement of the excitation electric field and increased focal intensity, thus led to the detection of more fine processes and dendritic spines. For functional imaging of dendritic and synaptic activity in the mouse primary visual cortex, the improved imaging performance enabled calcium and glutamate transients to be detected with higher SNR, which led to accurate characterization of their orientation-tuning properties. The signal improvement should also lead to better performance of demixing methods^43^ that can separate, in the Bessel images, laterally overlapping dendrites and synapses with distinct activity patterns. Therefore, by using the aberration-corrected Bessel focus for two-photon fluorescence excitation, a 2PFM can now image at both high spatial and high temporal resolution over three dimensions.

## Methods

### Animal use

All animal experiments were conducted according to the National Institutes of Health guidelines for animal research. Procedures and protocols on mice and zebrafish were approved by the Institutional Animal Care and Use Committee at the University of California, Berkeley (AUP-2020-06-13343).

### AO Bessel focus scanning 2PFM

The AO Bessel focus scanning 2PFM (Supplementary Fig. 1 and Supplementary Fig. 12) was built upon a homebuilt 2PFM system with a pupil-segmentation AO method implemented, as described in detail previously^17^. Briefly, a 940-nm femtosecond laser output (Insight DeepSee, Spectral Physics) was attenuated with a Pockel cell and then expanded 2× by a beam expander (BE). A half-wave plate was used to rotate the polarization of the laser so that its wavefront was effectively modulated by a reflective phase-only liquid-crystal spatial light modulator (SLM1, 1920 × 1152 Spatial Light Modulator, HSP1920, Meadowlark Optics). A custom LabVIEW^®^ program was used to acquire the two-photon images. In Gaussian mode (Supplementary Fig. 1a), a flat phase pattern was applied to SLM1, which simply acted as a reflective mirror. In Bessel mode (Supplementary Fig. 1b), a concentric binary phase grating (for Bessel focus generation without AO; period = 22.5 pixels/cycle, with alternate 0 and π phase shifts) or a focal-plane wavefront pattern for aberration-corrected Bessel focus was applied to SLM1. In addition, a lens (L1, focal length: 200 mm) was introduced to the beam path to generate a ring illumination pattern, which was then filtered by a transmissive annular mask (inner diameter = 1.015 mm, outer diameter = 1.2 mm; Photo Sciences, Inc.) to block spurious orders and undiffracted light. The surface of the mask was relayed onto two galvanometers (Galvo X and Y, 3-mm beam aperture; model 6215H; Cambridge Technology Inc.) by a pair of relay lenses (L2 and L3, 750 and 350 mm focal lengths, respectively). The galvanometers were optically conjugated to each other by two scan lenses (telecentric f-theta lenses with 30 mm focal length, Special Optics). Another pair of telecentric f-theta scan lenses (L4 and L5, 30 and 150 mm focal lengths, respectively) expanded the laser 5× and conjugated the Galvos to another reflective phase-only liquid-crystal spatial light modulator (SLM2, 512 × 512 Spatial Light Modulator, HSP512, Meadowlark). SLM2 was conjugated to the back pupil plane of a water-dipping microscope objective (Nikon, 25×, 1.05 NA, 2 mm WD) by another pair of telecentric f-theta scan lenses (L6 and L7, 120 and 240 mm focal lengths, respectively). For ease of alignment, both SLM1 and the annular mask were mounted on 3D translation stages and positioned to center along the optical axes of SLM2 and the objective. For aberration-corrected Gaussian focus scanning, SLM2 was used for indirect pupil-segmentation-based wavefront sensing and pupil-plane AO correction. For AO Bessel focus scanning, we derived the focal-plane corrective pattern for Bessel foci from the pupil-plane corrective pattern for Gaussian foci, following the procedure described in Fig. 1 and Supplementary Fig. 2, and applied the focal-plane wavefront pattern to SLM1 and a flat phase pattern to SLM2 to generate an aberration-corrected Bessel focus within biological samples. Fluorescence generated by two-photon excitation was collected in the epi-illumination direction, and detected using a photomultiplier tube (PMT; H7422-40, Hamamatsu).

In Fig. 1, we measured and corrected the optical system aberration. For all biological imaging data, system aberration was measured and corrected at the beginning of every experimental session, with all the “No AO” images obtained with an aberration-free microscope of diffraction-limited resolution, and the aberrations measured from biological samples originating only from the samples themselves. Consequently, the improvements observed from “No AO” to “AO” images resulted from the correction of sample-induced aberrations. For all experiments, post-objective laser powers were measured and adjusted to be the same for “No AO” and “AO” conditions. All experimental parameters are listed in Supplementary Table 1

### Pupil-segmentation-based indirect wavefront sensing and computation of the focal-plane corrective pattern for Bessel focus scanning

Pupil-segmentation-based indirect wavefront sensing with single segment illumination^17^ was used to measure both system and sample-induced optical aberrations. Briefly (Supplementary Fig. 2a), to obtain a diffraction-limited excitation focus similar to that observed in the ideal immersion medium where all rays intersect and constructively interfere at the focus, we need to determine the displacement and phase shift of individual beamlets when they propagate through an aberrating sample, where optical aberrations lead to an enlarged focal spot and decreased focal intensity. To measure the displacement of individual beamlets, we divided the objective back pupil into 5 × 5 segments, illuminated only one segment at a time, and took a two-photon fluorescence excitation image by scanning the resulting beamlet over an isolated fluorescent bead or cell body. Repeating this process for all pupil segments generated 25 images of the bead or cell body. We then localized the bead/cell body positions and compared them to a reference position, from which we obtained the phase gradient for each pupil segment. Finally, we computationally reconstructed the corrective wavefront assuming a spatially continuous aberration. Applying the corrective wavefront to SLM2, which was conjugated to objective pupil plane, fully recovered diffraction-limited Gaussian focus.

To obtain the corrective pattern on the focal plane for generating an aberration-free Bessel focus, we carried out the following steps (Supplementary Fig. 2b). First, we truncated the measured corrective wavefront into a circular pattern corresponding to the back pupil of the microscope objective, and decomposed the truncated pattern into the first 55 Zernike modes. Fitting the segmented wavefront with Zernike modes before propagating the phase through annular mask allowed us to remove defocus, tip, and tilt from the pupil corrective wavefront. Removing defocus prevents the generation of a Bessel focus with a skewed axial intensity profile (Supplementary Fig. 2d). Removing tip and tilt from the pupil corrective wavefront prevented spatial deviation between the annular illumination generated by the Bessel SLM and the annular mask, so that the illumination light can pass through the annular mask without being blocked. As shown in Fig. 2 and Supplementary Note, defocus, tip, and tilt do not degrade Bessel PSF, therefore removing these Zernike modes does not impair its imaging performance. Furthermore, the measured pupil corrective wavefront was made of flat segments with discontinuous wavefront gradients. Fourier transforming this wavefront within the annulus directly without fitting would lead to artifactual errors in the focal AO pattern. Then, we applied the annular mask (after adjustment for magnification) to this corrective wavefront and calculated the complex electric field corresponding to its Fourier transform. The phase pattern of this Fourier transform was then applied to SLM1, which was conjugated to the focal plane, to generate an aberration-corrected Bessel focus. We validated this approach computationally by forward propagating the phase-only corrective wavefront pattern to the pupil plane via an inverse Fourier transform, assuming a uniform amplitude profile. The resulting optical field intensity and phase distributions successfully replicated the expected corrective wavefront within the annular mask (Supplementary Fig. 2c). The MATLAB code for all steps is provided in Supplementary Software.

In order to achieve an axially symmetric intensity distribution for the Bessel focus, we removed defocus from the measured pupil-plane corrective wavefront prior to computing the focal-plane wavefront. Supplementary Fig. 2d plotted the axial signal profiles of a 2-µm-diameter fluorescent bead imaged by Bessel foci (NA = 0.4, FWHM = 43 µm) before system aberration correction, after correction but without removing defocus, and after correction and removal of defocus mode. Both aberration-corrected Bessel foci led to an increase in the signal intensity of the bead, whereas the removal of defocus generated a more axially symmetric signal profile.

### Mouse preparation

*Thy1*-GFP mice (males, >2months old) were used for morphological imaging. Wild-type mice (C57BL/6J, females or males, >2 months old) were used for in vivo functional imaging. The mice were housed in an animal facility at UC Berkeley campus with 12 light/12 dark cycle, ambient temperature between 20 and 26 °C, and humidity between 40 and 60%. The procedures of cranial window implantation and virus injection (for wild-type mice only) have been described previously^26^. Briefly, mice were anaesthetized with 1–2% isoflurane by volume in O_2_ and given the analgesic buprenorphine subcutaneously (0.3 mg per kg of body weight). A 3-mm craniotomy was then created over the V1 region of mice with dura left intact. Virus injection was performed using a glass pipette beveled at 45° with a 15–20-μm opening and back-filled with mineral oil. A fitted plunger controlled by a hydraulic manipulator (Narashige, MO10) was inserted into the glass pipette, which was used to inject the viral solution into the cortex at 200 and 350 µm below pia. Sparse expression of GCaMP6s, GCaMP7s, and iGluSnFR-A184S was achieved by injecting a 20 nl 1:1 mixture of diluted AAV2/1-syn-Cre virus (original titer: 10^12^ infectious units per ml, diluted 3000-fold in phosphate-buffered saline) and Cre-dependent GCaMP6s/GCaMP7s/iGluSnFR-A184S virus (AAV2/1.syn.Flex.GCaMP6s, 8 × 10^11^ infectious units per ml; AAV2/1-syn-flex-WPRE-jGCaMP7s, 1.6 × 10^13^ infectious units per ml, pAAV-CAG-FLEX.SF-iGluSnFR.A184S, 1.1 × 10^13^ infectious units per ml) per injection depth. The pipette was kept at each depth for about 1 min and remained in the brain for 5 min after virus injection before being pulled out. Two-micrometer-diameter carboxylate-modified fluorescent microspheres (F-8826, Invitrogen) diluted in saline were then applied on the cortical surface for later measurement of cranial-window-induced aberrations. A cranial window made of a single glass coverslip (Fisher Scientific, No. 1.5) was embedded in the craniotomy and sealed in place with Vetbond. A titanium headpost was then attached to the skull with cyanoacrylate glue and dental acrylic. In vivo imaging was carried out after 4 weeks of expression and 3 days of habituation for head fixation while awake. All imaging experiments were carried out on headfixed awake mice.

### Zebrafish preparation

Transgenic zebrafish *Tg(isl1:GFP)* was a gift from Dr. David Schoppik. Treatment of phenylthiourea was applied from 1 day post fertilization (dpf) to prevent pigmentation. At 5 dpf, GFP-positive fish were mounted dorsally onto a glass-bottomed petri dish (P50G-1.5-14-F, MatTek) with 1.4% agarose. Two-micrometer-diameter fluorescent beads (F8826, Invitrogen) were micro-injected into the brain with borosilicate pipette (#30-30-0, FHC Inc.). Zebrafish was immobilized with tricaine during image.

### Visual stimulation

Visual stimuli were delivered via a blue LED light source (450–495 nm, SugarCUBE) and back projected on a screen made of Teflon film (McMaster-Carr) using a custom-modified projector. The screen was positioned 17 cm from the right eye, covering 75° × 75° of visual space and oriented at ~40° to the long axis of the animal. The visual stimulation was presented as full-field drifting gratings towards 12 directions (0°–330° at 30° increments) in pseudorandom sequences. Gratings were of 100% contrast and 0.07 cycles per degree, moving at a speed of 26° s^−1^ (2-Hz temporal frequency). Each stimulus lasted 8 s (2-s blank, 4-s drifting grating, and 2-s blank) in Gaussian mode (frame rate = 3.3 Hz) and 12 s (4-s blank, 6-s drifting grating, and 2-s blank) in Bessel mode (frame rate = 1.5 Hz). Ten trials were repeated for each measurement and the trial-averaged results were presented.

### Image processing, analysis, and statistics

Imaging data were processed with Fiji^44^ and custom codes written in MATLAB^®^. Gaussian images were registered with an iterative cross-correlation-based registration algorithm while Bessel images were registered with non-rigid motion correction (NoRMCorre)^45^. Gaussian and Bessel images were not registered to each other. Morphological Bessel images, measured without as well as with AO, were also deconvoluted using the diffraction-limited Bessel PSF calculated from a Richard & Wolf model^46^. We found that deconvolution improved the brightness, SNR, and for calcium activity measurements, calcium transient detection of both “No AO” and “AO” images (Supplementary Fig. 11). However, more substantial improvements by deconvolution were observed in “AO” images, and deconvolution alone on “No AO” images did not provide the signal and sensitivity improvements offered by AO correction. For functional imaging analysis, no deconvolution was applied. ROIs were selected and outlined manually. The averaged fluorescence signal within the ROI (~2 µm in diameter) was extracted from each frame to generate the temporal calcium and glutamate activity profiles. For sensory-evoked activity studies, the basal fluorescence *F*_0_ was defined as the averaged fluorescence signal across 2 or 4 s of blank stimuli presented prior to the presentation of drifting-grating stimuli for Gaussian and Bessel images, respectively. Responses *R* evoked by visual stimuli were defined as the averaged ∆*F/F*_0_ across 4 and 6 s of drifting-grating stimulus presentation for Gaussian and Bessel images, respectively. A response with ∆*F/F*_0_ > 20% was considered as a calcium transient, while a threshold of >5% was used to define glutamate responses. Drifting-grating-evoked responses were defined as orientation-selective if the responses across the drifting-grating stimuli were significantly different (one-way ANOVA, *p* < 0.05). Assuming a normal distribution of *R* and equal variance across grating direction *θ*, the response evoked by drifting gratings of direction *θ* can be fitted with a bimodal Gaussian function^47^:

1 $$R\left(\theta \right)={R}_{{{\mbox{offset}}}}+{R}_{{{\mbox{pref}}}}{\mathrm e}^{-\frac{{{\mbox{ang}}}{({\theta -\theta }_{{{\mbox{pref}}}})}^{2}}{2{\sigma }^{2}}}+{R}_{{{\mbox{oppo}}}}{\mathrm e}^{-\frac{{{\mbox{ang}}}{({\theta -\theta }_{{{\mbox{pref}}}}+180^\circ )}^{2}}{2{\sigma }^{2}}}$$

where ${R}_{{{\mbox{offset}}}}$ is a contant offset, ${R}_{{{\mbox{pref}}}}$ and ${R}_{{{\mbox{oppo}}}}$ are the responses at the preferred grating drifting angle ${\theta }_{{{\mbox{pref}}}}$ and ${\theta }_{{{\mbox{pref}}}}-180^\circ$, respectively. The global orientation-selectivity index (gOSI) was defined as

2 $${{\mbox{gOSI}}}=\frac{{\sum }_{k}R\left({\theta }_{k}\right){\mathrm e}^{i2{\theta }_{k}}}{{\sum }_{k}R\left({\theta }_{k}\right)}$$

### Reproducibility

Optical system aberration corrections were performed for the Gaussian and Bessel foci before all imaging sessions and the measurements for Fig. 1b and  d were repeatedly four times in different imaging sessions. The measurements of aberrated Gaussian and Bessel PSFs with different Zernike modes (Fig. 2b–d) were measured four times within a single imaging session and results reproduced in two imaging sessions. The imaging of *Thy1*-GFP mice at different depths were repeated at least ten times with eight mice, with representative images shown in Figs. 3d, f, and Supplementary Figs. 5a, c–e, 7c, d and 8a, b. The imaging of zebrafish larval (Supplementary Fig. 9d, f) were repeated twice. The imaging of calcium signaling in the mouse primary visual cortex (V1) were repeated three times (Fig. 4b, d–f) for visual-evoked responses with different mice and two times (Figs. 10a, d, e, 11a–c, e–h) for spontaneous activity with a mouse. The imaging of glutamate signaling (Fig. 5) in V1 was repeated two times with two mice.

### Reporting summary

Further information on research design is available in the Nature Research Reporting Summary linked to this article.

## Supplementary information

Supplementary Information

Reporting Summary

Description of Additional Supplementary Files

Supplementary Software

Supplementary Movie 1

Supplementary Movie 2

Supplementary Movie 3

Supplementary Movie 4

Supplementary Movie 5

Supplementary Movie 6

## Data Availability

The authors declare that data supporting the findings of this study are available within the paper and its supplementary information files. Source data are provided with this paper.

## Source data

Source Data

## Code availability

MATLAB^®^ codes used to generate the AO-corrected Bessel phase patterns, the PSF simulation for non-circular symmetric aberrations are included in Supplementary Software. They can also be found at https://github.com/JiLabUCBerkeley/AOBessel, together with the uncompressed Supplementary Videos.
