# Supplementary material for: In vivo volumetric imaging of calcium and glutamate activity at synapses with high spatiotemporal resolution
Source: Nat Commun. 2021 Nov 16;12:6630. doi: 10.1038/s41467-021-26965-7 (PMC8595604; doi:10.1038/s41467-021-26965-7)
Supplement: Supplementary file 1 — Supplementary Information [file 41467_2021_26965_MOESM1_ESM.pdf]

Supplementary information

In vivo volumetric imaging of calcium and glutamate activity at synapses with high spatiotemporal resolution

Wei Chen[1], Ryan G Natan[1], Yuhan Yang[1], Shih-Wei Chou[2], Qinrong Zhang[1], Ehud Y Isacoff[2,3,4], Na Ji[1,2,3,4,*]

[1]Department of Physics, University of California, Berkeley, California, USA, 97420.

[2]Department of Molecular and Cell Biology, University of California, Berkeley, California, USA, 94720.

[3]Helen Wills Neuroscience Institute, University of California, Berkeley, California, USA, 94720.

[4]Molecular Biophysics and Integrated Bioimaging Division, Lawrence Berkeley National Laboratory, Berkeley, CA, USA, 94720.

*To whom correspondence should be addressed:

Na Ji, Ph.D.

Email: jina@berkeley.edu

Supplementary Note 1. Amplitude modulation of optical field within the illumination annulus at the pupil plane diminishes pupil AO correction efficiency for Bessel focus

We investigated why pupil AO with a phase-only SLM (SLM2 in **Fig. 1a**) performed poorly in recovering image quality for Bessel focus scanning 2PFM. We first compared how uncorrected residual aberrations at the objective pupil plane affected Gaussian versus Bessel foci. After correcting for optical system aberrations, we applied an astigmatism pattern with 2π amplitude onto SLM2 and then used our pupil-segmentation AO method to correct it. From the applied astigmatism pattern and the corrective wavefront, we obtained the residual aberrations in the pupil plane (**Supplementary Fig. 3a**). For Gaussian focus, the astigmatism pattern reduced the signal of a 2-μm-diameter bead to 32%. After AO correction, we recovered 94% of the aberration-free signal (**Supplementary Fig. 3b**), with the 6% signal drop attributed to the residual aberrations. For Bessel focus, the astigmatism pattern reduced the signal of a 2-μm-diameter bead to 36%. After the same pupil AO correction, 85% of the aberration-free signal was recovered, lower than the 94% signal recovery for the Gaussian focus (**Supplementary Fig. 3d**). This decrease was caused by the Bessel focus being more affected by the residual astigmatism (**Supplementary Fig. 3c**) than the Gaussian focus, as we described previously (**Fig. 2**). Residual aberrations in pupil AO correction of Bessel focus therefore do not explain the much poorer performance observed in experiments on both system and sample-induced aberrations,

In the above example, the astigmatism pattern was applied to the phase-only SLM2, which only perturbed the phase, but not the amplitude of the electric field at the pupil plane. In typical experiments, distortion of wavefront occurs throughout the excitation light path for both system and sample-induced aberrations. This leads to perturbation on both the phase and the amplitude profiles of the electric field. With a phase-only SLM, one could only correct the phase but not the amplitude distortion. We investigated how the uncorrected amplitude perturbation affected Gaussian and Bessel foci using numerical simulation (**Supplementary Fig. 4**). Starting with a perfect wavefront (uniform amplitude and phase), we introduced the same astigmatism pattern at 200 mm away from the pupil plane, which, in addition to causing phase aberrations at the pupil plane, also led to amplitude distortions at the pupil plane for both Gaussian (**Supplementary Fig. 4a**) and Bessel (**Supplementary Fig. 4c**) excitation light. We then calculated the axial 2PFM PSFs for the ideal case (i.e., no distortion in either amplitude or phase) as well as for the phase-corrected wavefront (i.e., no distortion in phase, but with distortion in amplitude). Whereas for Gaussian focus, minimal (2.1%) degradation was observed due to the uncorrected amplitude distortion (**Supplementary Fig. 4b**), the uncorrected amplitude distortion led to only 33.5% recovery for Bessel PSF (**Supplementary Fig. 4d**), even though phase distortions were fully corrected at the pupil plane for both cases. In other words, with a phase-only SLM at the pupil plane, aberrations away from the pupil plane cause un-corrected distortions in the amplitude profiles of for both Gaussian and Bessel excitation at the pupil plane. Likely due to the much smaller illumination profile of Bessel focus, such amplitude distortions lead to more severe decrease in Bessel focal intensity.

In contrast, with focal AO, the phase-only SLM1 placed at a plane conjugate to the focal plane can introduce both amplitude and intensity corrections for the annular illumination pattern at the pupil plane. For the same example aberration, the focal corrective wavefront led to a complementary amplitude distortion to that caused by aberration (**Supplementary Fig. 4e**, patterns to the left of the equality sign), which reduced the final amplitude distortion after focal AO correction (**Supplementary Fig. 4e**, pattern to the right of the equality sign). Calculating the PSF after focal AO correction, we found that 91.9% of the signal was recovered (**Supplementary Fig. 4f**), substantially higher than pupil AO correction. In summary, focal AO overperforms pupil AO in correcting aberrations experienced by Bessel focus because a phase-only correction in the focal plane can correct both phase and amplitude distortions of the excitation light in the pupil plane, while a phase-only correction in the pupil plane is unable to correct amplitude distortion, which causes larger signal degradation for Bessel foci.

Supplementary Note 2. Aberration correction restores diffraction-limited resolution for volumetric imaging of zebrafish larval *in vivo*

In addition to the mouse brain, we also tested our method in the brain of zebrafish larvae mounted dorsally with 1.4% agarose, because zebrafish larva is another model organism often investigated with optical microscopy. At 120 hours post fertilization, we injected 2-μm-diameter red fluorescent beads into a transgenic zebrafish embryo (*Tg(isl1:GFP)*) densely expressing GFP in cranial motor neurons under the control of the islet-1 promoter (1, 2) (**Supplementary Fig. 9a**). We then measured the tissue-induced aberration using an isolated fluorescent bead at Z = 130 μm within the imaging field (arrow, **Supplementary Fig. 9b**) and obtained corrective wavefront patterns for Gaussian and Bessel foci (**Supplementary Fig. 9c**). We observed severe wavefront distortion in the hindbrain of the zebrafish larvae. Without aberration correction, images of motor neurons and their neuronal processes, especially their axonal projections (XY: 128 ×128 μm^2; Z: 80-140 μm, **Supplementary Fig. 9e**) were dimmer with many processes unresolved, which became much brighter and clearly resolvable after AO correction for both Gaussian (**Supplementary Fig. 9d**) and Bessel images (**Supplementary Figs. 9f,g**).

Samples with high and varying surface curvatures, such as zebrafish, may generate aberrations that are highly spatially variable (3). This may reduce the effectiveness of AO correction for structures more distant from the location of aberration measurement. For our sample and imaging area, however, we observed global improvements of image quality across the entire imaging volume of 128×128×60 μm^3, as indicated by the line intensity profiles without and with AO (90-μm-long dashed purple lines, in **Supplementary Figs. 9d,f**) , where the signal and resolution improvements were evident for both Gaussian and Bessel images (**Supplementary Fig. 9g,** up to 2.7× and 2.5× signal improvements for Gaussian and Bessel images, respectively). The global improvements within this volume were also reflected in the spatial frequency domain (**Supplementary Fig. 9h**), with the post-correction Bessel image contained more high spatial frequency components, enabling high-resolution 3D morphological measurement of zebrafish larvae up to the diffraction limit.

Supplementary Note 3. AO enhances detection of spontaneous calcium transients in volumetric imaging of dendritic spines in the awake mouse V1 by Bessel focus scanning *in vivo*

Since its invention, 2PFM has been widely used to study the functional activity of neurons (4, 5). Combining 2PFM with Bessel focus scanning, we were able to substantially increase the volumetric functional imaging throughput (6-8). Here, we evaluated how aberration correction would benefit calcium imaging of neurons expressing the genetically encoded calcium indicator GCaMP6s (9) (**Supplementary Fig. 10**). Using viral transduction strategies, we sparsely expressed GCaMP6s in the mouse V1. We measured the wavefront aberration experienced at the cell body of a GCaMP6-expressing neuron at 230 µm depth. With AO correction (corrective wavefronts, **Supplementary Fig. 10b**), fluorescence signal of the cell body and nearby dendrites in the Gaussian images increased about 1.6× and 3.5×, respectively (**Supplementary Fig. 10a**). We then took 3D Gaussian image stacks of its dendritic branches within a 128 × 100 × 60 µm^3 volume (**Supplementary Figs. 10c,d**), as well as 2D Bessel focus scanning images of the same volume without and with AO correction (**Supplementary Fig. 10e**). Because this neuron was spontaneously active, we selectively averaged the frames without elevated somatic calcium transients. For both Gaussian image stacks (**Supplementary Fig. 10d**) and Bessel frames (**Supplementary Fig. 10e**), AO increased the basal fluorescence signal (F_0) of soma, dendrites, and spines, as well as improved spatial resolution to give rise to sharper images of dendritic spines. For Bessel images, we observed 1.3×, 2× and up to 4× signal increases for soma, dendrites, and spines, respectively (**Supplementary Fig. 10f**), consistent with the morphological imaging results of the Thy1-GFP line M mice.

Furthermore, we compared the time series obtained by Bessel focus scanning of the same volume at 2 Hz for 400 s, without and with AO correction, during which spontaneous activity was captured from dendrites and dendritic spines in the entire volume. As shown in example fluorescence signal traces for 4 spines (**Supplementary Fig. 10g**), in addition to increasing their basal fluorescence signal F_0, aberration correction also improved the detection sensitivity of spontaneous calcium transients from these spines (**Supplementary Movie 1**). The high-throughput of the Bessel focus scanning method enabled us to measure spontaneous calcium transients from 140 spines from different depths and dendritic branches simultaneously, in contrast to Gaussian imaging data where only structures within a thin optical section were captured (**Supplementary Movie 2**). Across the population of spines, we observed on average a 1.6× increase in F_0, and an up to 4× increase for spines that were unresolvable before AO correction (**Supplementary Fig. 10h**). The increase in basal fluorescence F_0 also led to improvement in signal-to-noise ratio (SNR) defined as the ratio of the time-averaged F_0 and its standard deviation σ (**Supplementary Fig. 10i**). Consequently, more calcium transient events, defined as episodes of elevated fluorescence signals with $(F\text{-}F_0)>3\sigma$, were detected after AO correction (**Supplementary Fig. 10j**, 9.9±0.3 events without AO versus 29.3±0.7 events with AO, 140 spines, paired t-test, $p<0.001$).

Supplementary Note 4. Numerical simulation of Bessel PSF perturbed by noncircularly symmetric aberrations

To calculate the point spread function (PSF) of an imaging system for incident electric fields with noncircularly symmetric wavefront aberrations (e.g., astigmatism and trefoil), we developed a numerical model based on the vector diffraction theory derived by Novotny and Hecht (10). Here, a microscope objective transforms the electric field at its back pupil plane, $\mathrm{E_{inc}}$, into an electric field at the reference spherical surface $\mathbf{E}_\infty$ (**Supplementary Fig. 13a**). Assuming the incident electric field is linearly polarized along the x-axis, $\mathbf{E}_\infty$ can be expressed as

$$\mathbf{E}_\infty(\theta,\phi) = \mathrm{E_{inc}}(\theta,\phi)\left[\cos\phi\,\boldsymbol{n}_\theta - \sin\phi\,\boldsymbol{n}_\phi\right]\sqrt{n_1/n_2}(\cos\theta)^{1/2}, \quad (1)$$

with the unit vectors $\boldsymbol{n}_\theta$ and $\boldsymbol{n}_\phi$ (of the spherical coordinate system shown in **Supplementary Fig. 13a**) representing p- and s-polarization, respectively. $\sqrt{n_1/n_2}(\cos\theta)^{1/2}$ is a consequence of the intensity law (11) which ensures energy conservation at the reference sphere interface with refractive index n_1 and n_2 on each side. Eq. (1) can be represented in Cartesian vector components $\left[\mathrm{E_x}\ \mathrm{E}_y\ \mathrm{E}_z\right]'$ as

$$\mathbf{E}_\infty(\theta,\phi) = \begin{bmatrix}\mathrm{E_x}\\ \mathrm{E_y}\\ \mathrm{E}_z\end{bmatrix} = \mathrm{E_{inc}}(\theta,\phi)\frac{1}{2}\begin{bmatrix}(1+\cos\theta-(1-\cos\theta)\cos2\phi)\\ -(1-\cos\theta)\sin2\phi\\ -2\cos\phi\sin\theta\end{bmatrix}\sqrt{n_1/n_2}(\cos\theta)^{1/2}. \quad (2)$$

To calculate the electric field near the focus of the objective, $\mathbf{E}_\infty$ is propagated to a plane located at *z* relative to the focus of an objective with a focal length of f, by integrating over θ and ϕ as

$$\mathbf{E}(\rho,\varphi,z) = \frac{ikfe^{-ikf}}{2\pi}\iint \mathbf{E}_\infty(\theta,\phi)e^{ikz\cos\theta}e^{ik\rho\sin\theta\cos(\phi-\varphi)}\sin\theta\, d\phi\, d\theta \quad (3)$$

where ρ,φ,z are cylindrical coordinates of the optical field around the focus.

From Eq. (3), we numerically calculated the optical field around the focus, including for the scenario where a phase perturbation with noncircular symmetry was included in the incident field $\mathrm{E_{inc}}$ (i.e., the phase of $\mathrm{E_{inc}}$ is dependent on azimuthal angle ϕ). To simulate the PSFs of Bessel-like foci, we also applied an annular mask to $\mathrm{E_{inc}}$, as shown in **Supplementary Fig. 13b**. The final Bessel PSF was calculated as the fourth power of the calculated focal field amplitude to reflect two-photon excitation. An example code used to calculate the Bessel 2PFM PSF distorted by astigmatism (Zernike coefficient =1) is provided in **Supplementary Software**.

Supplementary Note 5. A mathematical analysis demonstrating that ideal Bessel beam is only sensitive to Zernike aberration modes with azimuthal index $|m| > 1$

The aberration present in the incident wavefront, ψ, can be described as the summation of a series of orthogonal Zernike polynomials on the unit disk. In polar coordinates (see **Supplementary Fig. 14** for their geometric representations):

$$\psi(r,\theta) = \sum_{i=1}^{\infty} M_i\, Z_i(r,\theta), \quad (4)$$

where M_i is the coefficient that describes the amplitude of Zernike mode $Z_i(r,\theta)$. With $i = (n(n+2)+m)/2$, the Zernike modes are defined as:

$$Z_n^m(r,\theta) = \begin{cases} m < 0: & R_n^{-m}(r)\sin(-m\theta) \\ m = 0: & R_n^m(r) \\ m > 0: & R_n^m(r)\cos(m\theta) \end{cases} \tag{5}$$

$$R_n^m(r) = \sum_{s=0}^{(n-m)/2} \frac{(-1)^s (n-s)!}{s!((n+m)/2-s)!((n-m)/2-s)!} r^{n-2s}, \tag{6}$$

where the indices (n, m) are the radial index n and azimuthal index m, respectively.

Define an electric field of infinitely thin ring illumination and flat wavefront at the pupil plane of a microscope objective as:

$$u_0(r,\theta) = A_0\delta(r,r_0), \tag{7}$$

with $\delta(r,r_0)$ being 1 for $r = r_0$ and 0 for $r \neq r_0$.

With aberration, the electrical field at the pupil plane becomes

$$u(r,\theta) = A_0\delta(r,r_0)\exp[-j(2\pi Z_n^m(r,\theta))]. \tag{8}$$

The optical field v at the focal plane of an objective can be calculated by 2D Fourier transform as:

$$v(\rho,\phi) = A_0\iint dr d\theta r \exp[-j2\pi Z_n^m(r,\theta)]\exp[-j2\pi r\rho(\cos\theta\cos\phi + \sin\theta\sin\phi)]\delta(r,r_0). \tag{9}$$

Discussion:

(**a**) When $m = 0$, for instance, defocus Z_2^0 and spherical aberrations Z_4^0, Eq. (9) becomes

$$\begin{aligned} v(\rho,\phi)_{m=0} &= A_0\iint dr d\theta r \exp[-j2\pi R_n^0(r)]\exp[-j2\pi r\rho(\cos\theta\cos\phi + \sin\theta\sin\phi)]\delta(r,r_0) \\ &= A_0 \cdot \exp[-j2\pi R_n^0(r_0)] \cdot \int \exp[-j2\pi r_0\rho(\cos\theta\cos\phi + \sin\theta\sin\phi)]d\theta \\ &= A_0 \cdot \exp[-j2\pi R_n^0(r_0)] \cdot \int \exp[-j2\pi r_0\rho\cos(\theta-\phi)]d\theta. \end{aligned} \tag{10}$$

Based on the Bessel function identity

$$J_0(a) = \frac{1}{2\pi}\int \exp[-ja\cos(\theta-\phi)]d\theta \tag{11}$$

Eq. (10) becomes

$$v(\rho,\phi)_{m=0} = A_0 \cdot \exp[-j2\pi R_n^0(r_0)] \cdot J_0(2\pi r_0\rho), \tag{12}$$

Where J_0 is a Bessel function of the first kind, zero order. Therefore, aberrations with azimuthal frequency $m = 0$ only add a constant phase term and do not change the intensity profile of the Bessel focus at focal plane.

(b) When $m = 1$, for instance, coma Z_3^1 and tilt Z_1^1, Eq. (9) becomes:

$$v(\rho,\phi)_{m=1} = A_0 \iint dr d\theta r \exp[-j2\pi R_n^1(r)\cos\theta]\exp[-j2\pi r\rho(\cos\theta\cos\phi + \sin\theta\sin\phi)]\delta(r, r_0). \tag{13}$$

Switching to cartesian coordinates (**Supplementary Fig. 14**), we have:

$x = r_0 \cos\theta$, $y = r_0 \sin\theta$,

with $\delta(r, r_0)$ defining $(x, y) \in S, S: \sqrt{x^2 + y^2} = r_0$;

$f_x = \rho\cos\phi$, $f_y = \rho\sin\phi$,

Eq. (13) can be rewritten as:

$$\begin{aligned} v(f_x, f_y)_{m=1} &= A_0 \iint dx dy \exp[-j2\pi R_n^1(r_0)\frac{x}{r_0}]\exp[-j2\pi(f_x x + f_y y)] \\ &= A_0 \iint dx dy \exp[-j2\pi \frac{R_n^1(r_0)}{r_0} x]\exp[-j2\pi(f_x x + f_y y)] \\ &= A_0 \iint dx dy \exp\left\{-j2\pi\left[\left(f_x + \frac{R_n^1(r_0)}{r_0}\right)x + f_y y\right]\right\}, (x, y) \in S \end{aligned} \tag{14}$$

According to the shifting theorem of Fourier transform, the solution of Eq. (14) shifts the Fourier transform of the electrical field $u_0(x, y) = A_0, (x, y) \in S$ laterally in f_x direction by $\frac{R_n^1(r_0)}{r_0}$,

$$v\left(f_x - \frac{R_n^1(r_0)}{r_0}, f_y\right)_{m=1} = \Im\{u_0(x, y)\}, (x, y) \in S, \tag{15}$$

but does not change the focal intensity distribution described by $\Im\{u_0(x, y)\}$, the Fourier transform of the ideal ring illumination $u_0(x, y) = A_0, (x, y) \in S$. Similar results can be derived for $m = -1$, that the Bessel focus is laterally shifted in f_y direction by $\frac{R_n^1(r_0)}{r_0}$. Therefore, the only effect of Zernike aberration modes with azimuthal index $|m| = 1$ on ideal Bessel focus is to shift it laterally by $\frac{R_n^1(r_0)}{r_0}$.

(c) When $m > 1$, for instance, astigmatism Z_2^2 and trefoil Z_3^3, Eq. (9) becomes the Fourier transform of a complex function:

$$v(\rho,\phi)_{m>1} = A_0 \iint dr d\theta r \exp[-j2\pi R_n^m(r)\cos(m\theta)]\exp[-j2\pi r\rho(\cos\theta\cos\phi + \sin\theta\sin\phi)]\delta(r, r_0). \tag{16}$$

Based on the Binomial Expansion (12)

$$\cos(m\theta) = \sum_{k=0\,(\mathrm{even})}^{m} (-1)^{k/2} \binom{m}{k} r^{-m} x^{m-k} y^k. \tag{17}$$

Eq. (15) can be written in Cartesian coordinate as:

$$v(f_x, f_y)_{m>1} = A_0 \iint dx dy \exp[-j2\pi R_n^m(r_0) \cdot \sum_{k=0\,(\mathrm{even})}^{m} (-1)^{k/2} \binom{m}{k} r_0{}^{-m} x^{m-k} y^k]\exp[-j2\pi(f_x x + f_y y)] \quad ,$$

$$(x, y) \in S, S: \sqrt{x^2 + y^2} = r_0. \tag{18}$$

Even without solving the equation analytically, it becomes apparent that when $m > 1$, the coupled terms, $x^{m-k}y^k$, distort the focal intensity distribution away from that of an ideal Bessel beam. Similar results can be derived for $m < -1$.

In summary, the above analysis indicates that the intensity distribution of an ideal Bessel beam is insensitive to Zernike aberration modes with azimuthal index $|m| \leq 1$. This conclusion also applies effectively to the Bessel-like focus formed by an annular illumination of finite thickness, as employed in our study.

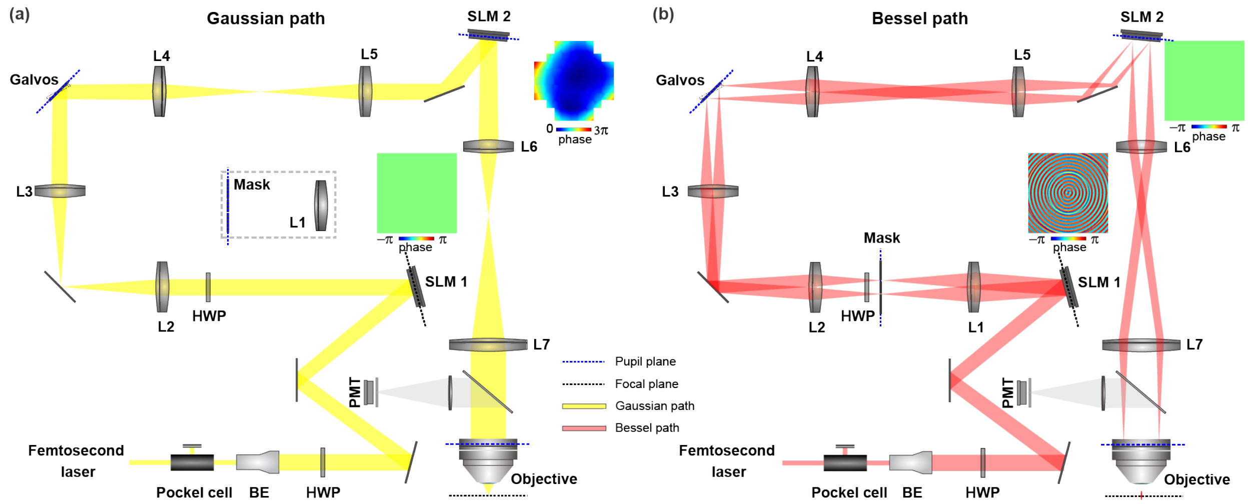

Supplementary Fig. 1. Schematics of AO Gaussian and Bessel focus scanning 2PFM light paths. See Methods for detail. (**a**) For AO correction of Gaussian focus, a flat phase pattern was applied to SLM1 and a pupil-plane corrective pattern was applied to SLM2. (**b**) For AO correction of Bessel focus, a lens (L1) and annular mask were placed into the beam path. A focal-plane wavefront pattern was applied to SLM1 to generate an annular illumination at the front focal plane of L1. After being filtered by the annular mask placed at the same plane, the annular illumination was conjugated to and reflected off the SLM2 (displaying a flat phase pattern), to generate an aberration-free Bessel focus. Dashed blue and black lines: planes conjugated to objective back pupil plane and objective focal plane, respectively.

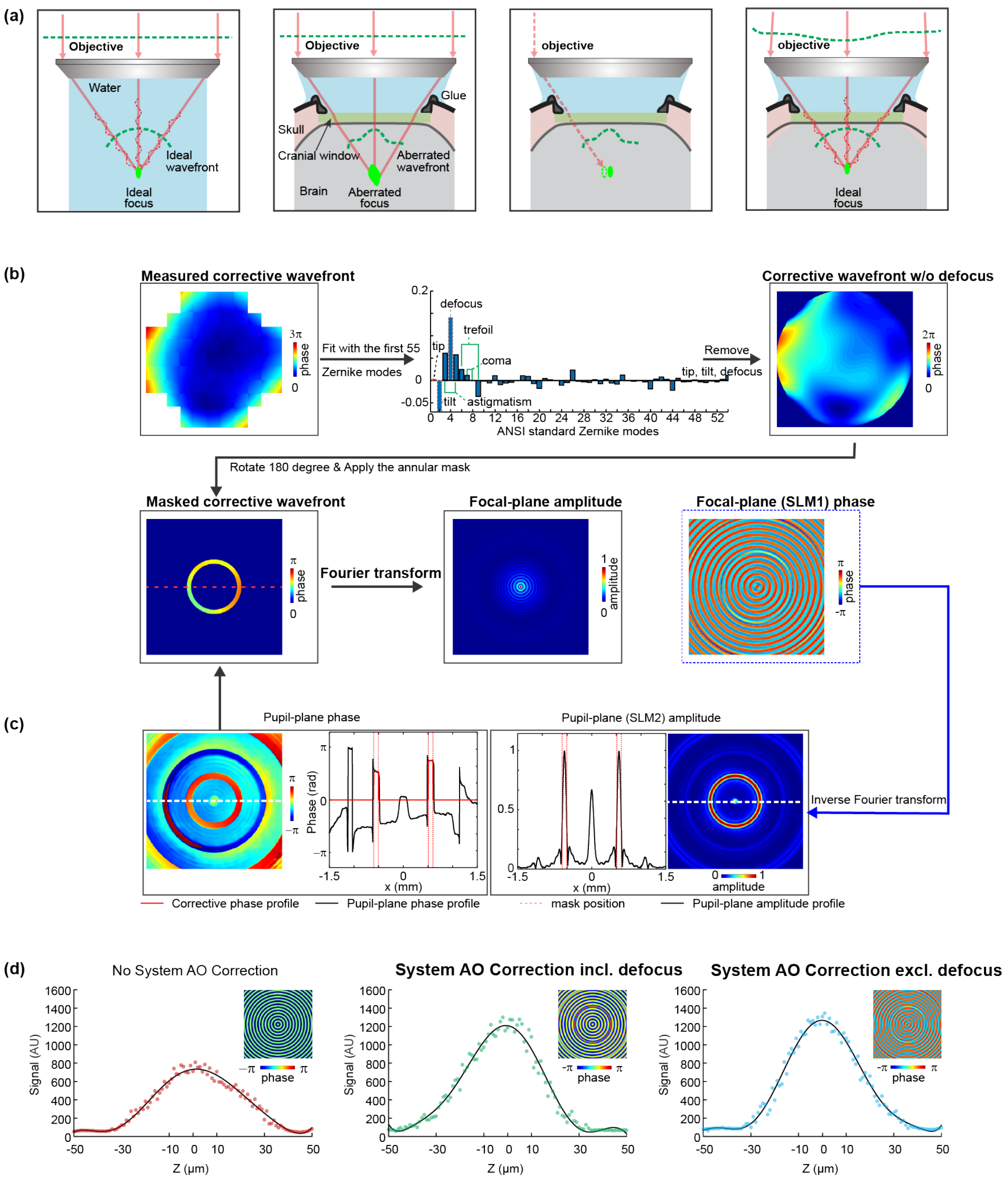

Supplementary Fig. 2 Step-by-step description of pupil-segmentation-based indirect wavefront sensing and computation of the focal-plane wavefront pattern for aberration-corrected Bessel focus. See Methods for details. (**a**) Schematics of pupil-segmentation-based indirect wavefront sensing with single segment illumination. (**b**) Steps to compute the focal-plane wavefront pattern for aberration-corrected Bessel focus: Fit the measured pupil-plane corrective wavefront with the first 55 Zernike modes; Remove tip, tilt, and defocus; from the other 52 Zernike modes, calculate a corrective wavefront; apply the annular mask (after adjustment for magnification) and calculate its Fourier transform; the phase pattern of this Fourier transform is applied to SLM1 as the focal-plane wavefront pattern for aberration-corrected Bessel focus. (**c**) Computational validation of the approach in **b**. Inverse Fourier transform of the focal-plane phase pattern results in optical field amplitude and phase distributions that replicate the expected corrective wavefront within the annular mask. MATLAB codes for **b** and **c** are provided in **Supplementary Software**.

(**d**) Removing defocus from the pupil-plane corrective wavefront prior to computational propagation leads to an axially symmetric intensity distribution for the Bessel focus: (From left to right) axial signal profiles of a 2-μm-diameter fluorescent bead excited by Bessel foci (NA=0.4, FWHM=43 μm) before system aberration correction, after correction but without removing defocus, and after correction and removal of defocus mode. Insets: phase patterns on SLM1. AU: arbitrary unit.

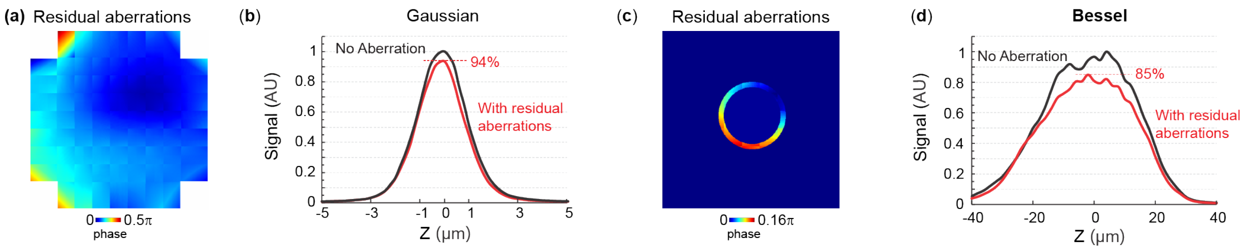

Supplementary Fig. 3. Effects of residual aberrations on Gaussian and Bessel foci. (**a**) Residual aberrations at the objective pupil plane after pupil AO correction of an astigmatism wavefront pattern applied to SLM2. (**b**) Axial signal profiles of a 2-µm-diameter bead measured without aberration and with residual aberrations in a, using Gaussian focus scanning 2PFM. (**c**) Residual aberrations (same as in **a**) within the illumination annulus. (**d**) Axial signal profiles of a 2-µm-diameter bead measured without aberration and with residual aberrations in a, using Bessel focus scanning 2PFM. AU: arbitrary unit.

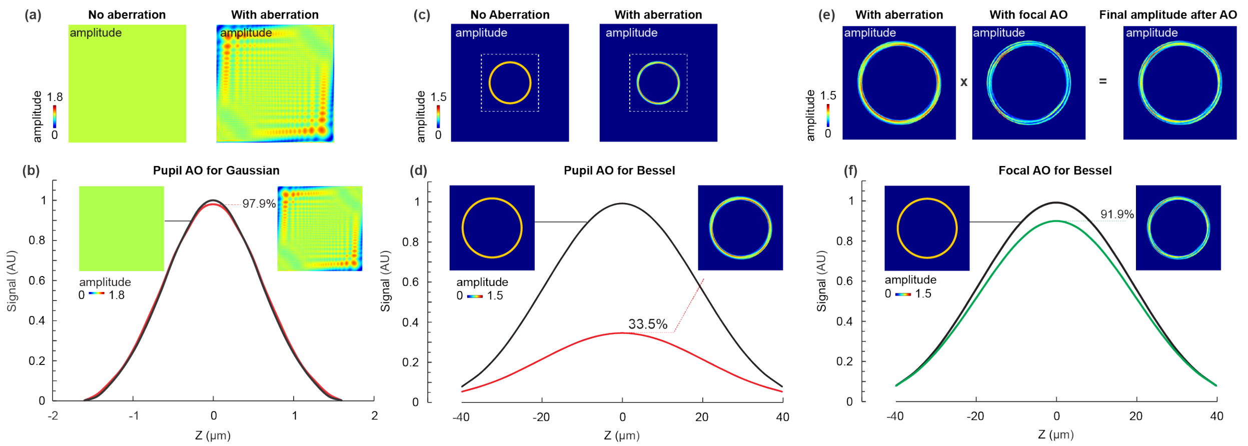

Supplementary Fig. 4. Effect of amplitude distortions on PSF for Gaussian and Bessel focus scanning 2PFM. (**a**, **c**) Numerically simulated amplitude profiles of Gaussian and Bessel illumination at the objective pupil plane without and with astigmatism introduced at Z=-200 mm away from the pupil plane. (**b**, **d**) Axial profiles of the ideal Gaussian and Bessel foci (black curves, flat amplitude and phase profiles) and the Bessel and Gaussian foci with only phase correction (red curves, distorted amplitude profile and flat phase profile). (**e**) Focal AO correction leads to a complementary amplitude profile to the aberration-induced amplitude profile, with the multiplication of the two amplitude profiles leading to a less distorted amplitude profile after focal AO correction. (**f**) Axial profiles of the ideal Bessel foci and the Bessel foci with focal AO correction (green curve, less distorted amplitude profile and flat phase profile). AU: arbitrary unit.

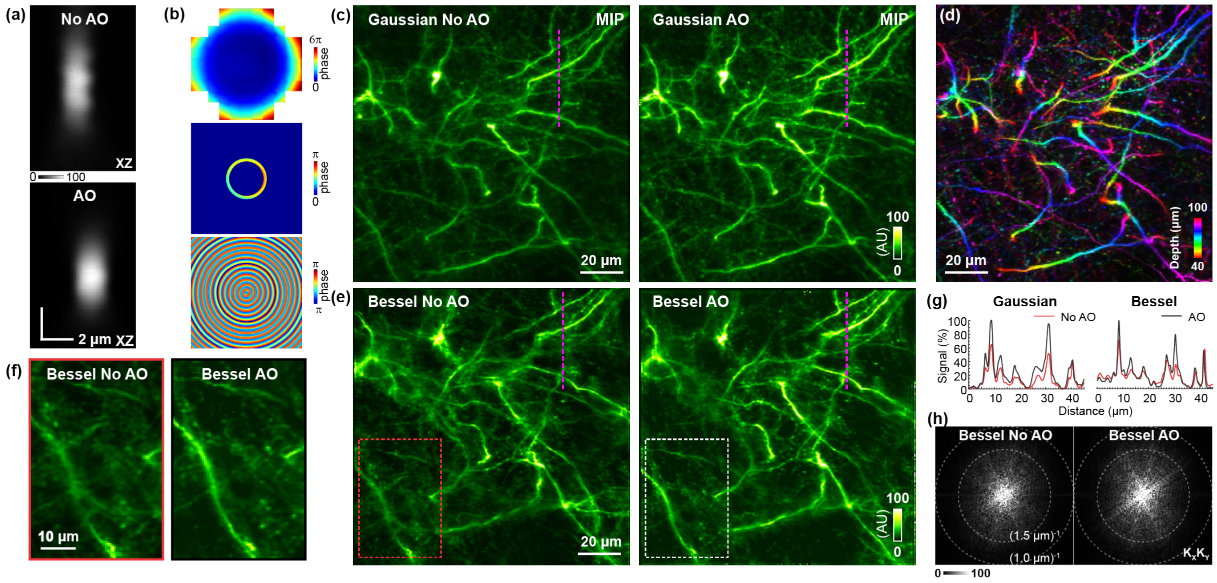

Supplementary Fig. 5. AO correction of cranial-window-induced aberrations for Gaussian and Bessel imaging in V1 of a Thy1-GFP mouse *in vivo*. (**a**) Axial images of a 2-µm-diameter fluorescent bead under cranial window, measured without and with AO correction of Gaussian focus. Aberration measurement was performed using this bead. (**b**) Wavefront on pupil plane/SLM2 for aberration correction of Gaussian focus, after transmitted through the annular mask, and the computed focal-plane pattern on SLM1 for aberration-corrected Bessel focus. (**c**) Mean intensity projections (MIP) of Gaussian imaging stacks from Z = 40 µm to Z = 100 µm without and with AO. (**d**) Gaussian imaging stack with AO color-coded by depth. (**e**) Bessel images of the same volume as in **d**, obtained without and with AO. (**f**) Zoomed-in views of the volume within dashed boxes in **e**. (**g**) Signal profiles of dendritic spines along the dashed purple lines in **c** and **e**. (**h**) Spectral power in the spatial frequency space (K_XK_Y) for images in **e** (pixel size: 0.5 µm). Post-objective powers: 27 mW for Gaussian and 62 mW for Bessel. Wavelength: 940 nm. AU: arbitrary unit.

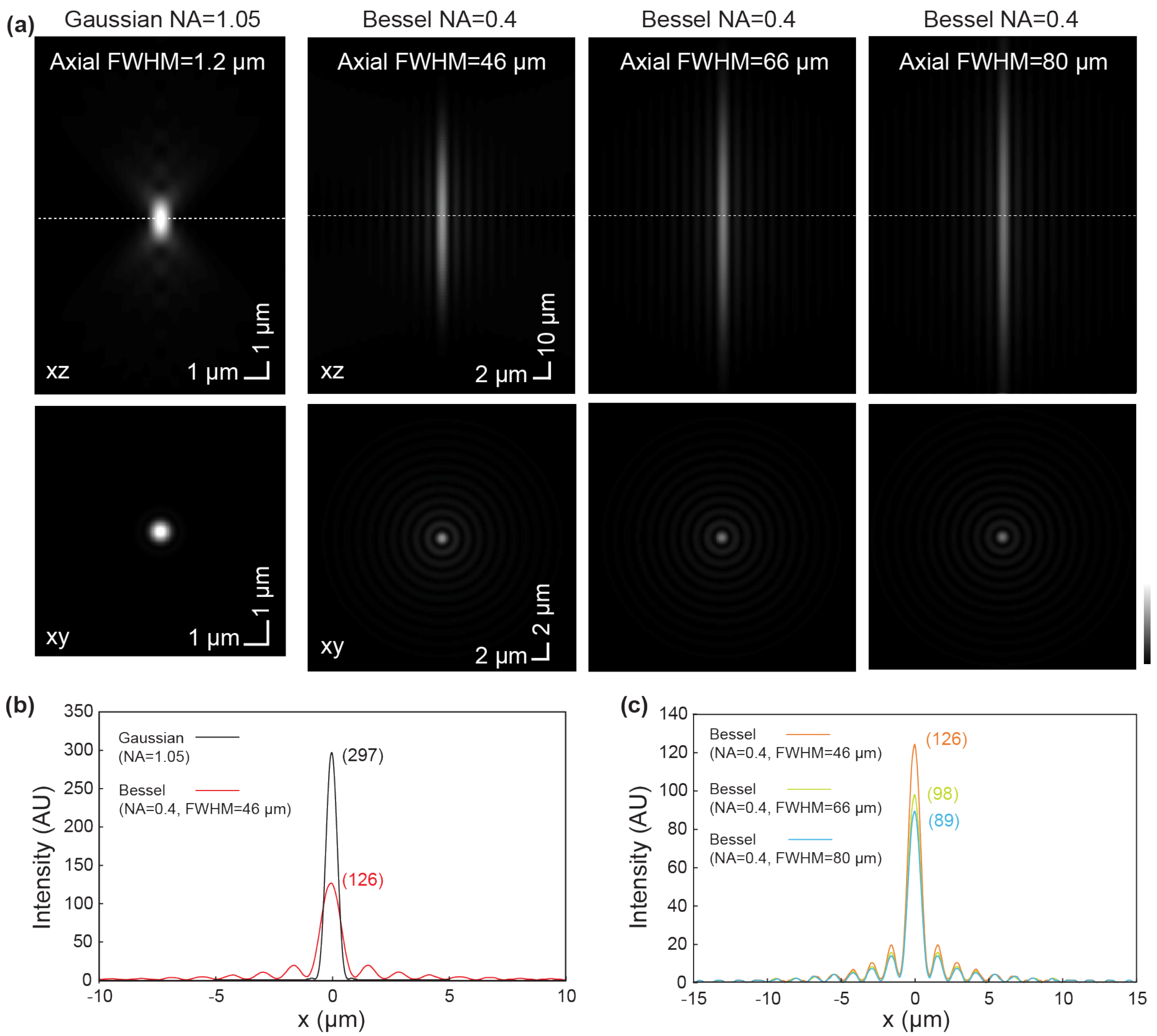

Supplementary Fig. 6. Simulated PSFs for Bessel and Gaussian foci. (**a**) Axial and lateral two-photon excitation PSFs (calculated as I^2) for the 1.05-NA Gaussian focus and 0.4-NA Bessel foci with axial FWHMs of 46 μm, 66 μm, and 80 μm, respectively. *I*: intensity of excitation light. All foci have the same post-objective power. Gamma corrections of γ=0.4 were applied to all PSFs to improve visibility of side rings. (**b**) Line profiles of the lateral one-photon PSFs (calculated as *I*) for 1.05-NA Gaussian focus and 0.4-NA Bessel focus with axial FWHMs of 46 μm, indicating that the peak focal intensity of the Bessel focus is 42% of that of the Gaussian focus. For a point object to reach similar signal level, laser power needs to increase by ~2× for the Bessel focus. For larger objects (e.g., cell bodies of neurons), smaller or even no power increase is needed to get the same signal, because the extended axial excitation by Bessel foci can compensate for the decrease in peak focal intensity. (**c**) Line profiles of the lateral one-photon PSFs of 0.4-NA Bessel foci with axial FWHMs of 46 μm, 66 μm, and 80 μm, respectively, indicating that longer Bessel foci have more energy distributed to the side rings and lower peak intensity. In **b** and **c**, numbers in paratheses indicate the peak intensity values. AU: arbitrary unit.

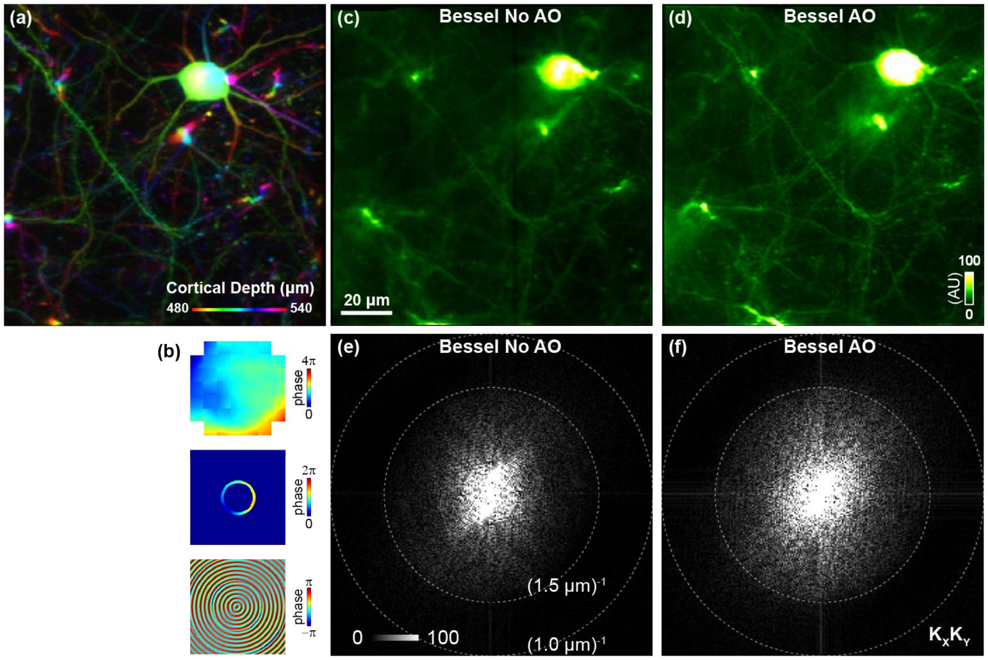

Supplementary Fig. 7. AO improves volumetric imaging of dendritic and synaptic morphology in layer 5 of Thy1-GFP mouse cortex *in vivo*. (**a**) A Gaussian imaging stack of a neuron and neurites located from Z = 480 µm to Z = 540 µm in V1 of a Thy1-GFP mouse, color-coded by depth. (**b**) Wavefront on pupil plane/SLM2 for aberration correction of Gaussian focus, after transmitted through the annular mask, and the computed focal-plane pattern on SLM1 for aberration-corrected Bessel focus. Gaussian corrective wavefront was measured based on the isolated soma body at Z = 500 µm shown in **a**. (**c,d**) Bessel images of the volume in **a** obtained without and with AO correction, respectively. (**e,f**) Spectral power in the spatial frequency space (K_XK_Y) for Bessel images in **c,d** (pixel size: 0.5 µm). Post-objective power: 116 mW for Gaussian; 102 mW for Bessel. Wavelength: 940 nm. AU: arbitrary unit.

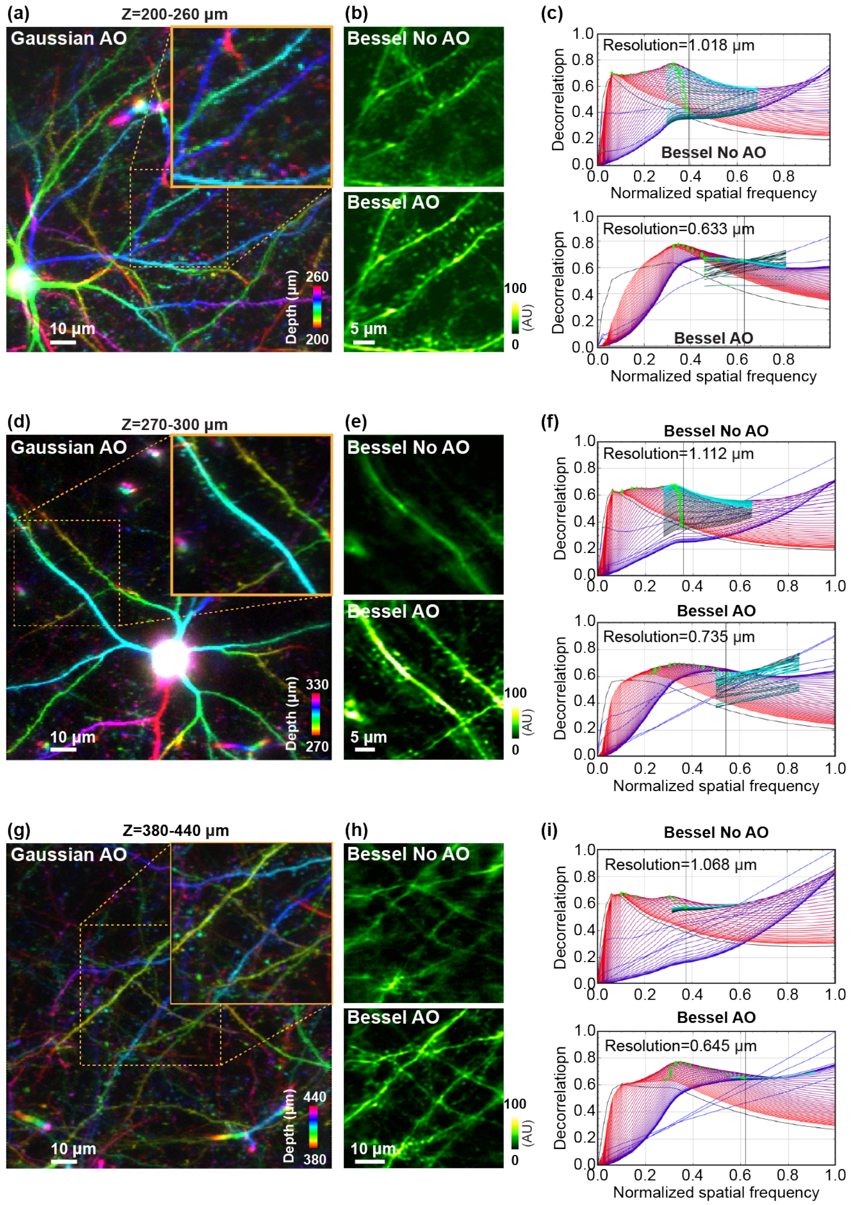

Supplementary Fig. 8. Resolution estimation of Bessel images without and with AO correction. (**a, d, g**) Colored-encoded depth projection of the volume acquired with Gaussian focus. Dashed box: FOV imaged with the Bessel focus. (**b, e, h**) Bessel images of the small FOVs in **a**, **d**, **g** without and with AO corrections. Pixel size: 0.2 µm. (**c, f, i**) Decorrelation analyses of the images in **b, e, h** with Ng=50, Nr=50. Vertical gray lines: locations of k_c. AU: arbitrary unit.

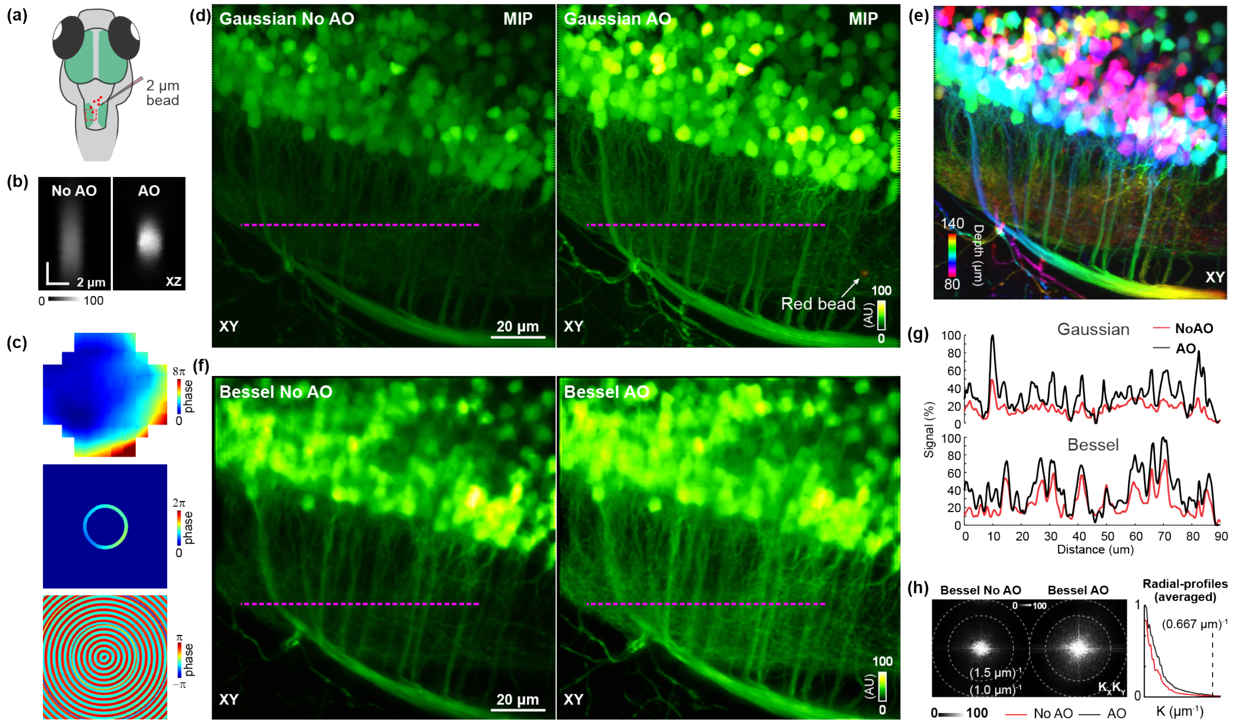

Supplementary Fig. 9. Aberration correction restores diffraction-limited resolution for volumetric imaging of zebrafish larval *in vivo*. (**a**) A schematic diagram of larval *isl1:GFP* transgenic zebrafish injected with 2-μm-diameter red fluorescent beads, on which the sample induced aberration was measured. (**b**) Axial images of the fluorescent bead measured without and with AO correction of Gaussian focus. (**c**) Wavefront on pupil plane/SLM2 for aberration correction of Gaussian focus, after transmitted through the annular mask, and the computed focal-plane pattern on SLM1 for aberration-corrected Bessel focus. (**d**) Mean intensity projections (MIP) of Gaussian imaging stacks of motor neurons and their axons from Z = 80 μm to Z = 140 μm measured without and with AO. (**e**) Gaussian imaging stack with AO color-coded by depth. (**f**) Bessel images of the same volume as in **d**, obtained without and with AO. (**g**) Signal profiles of neuropils along the dashed purple lines in **d** and **f**. (**h**) Spectral power in the spatial frequency space ($K_X K_Y$) for Bessel images in **e** (pixel size: 0.5 μm) and averaged radial profiles. Post-objective powers: 30 mW for Gaussian and 90 mW for Bessel. Wavelength: 940 nm. AU: arbitrary unit.

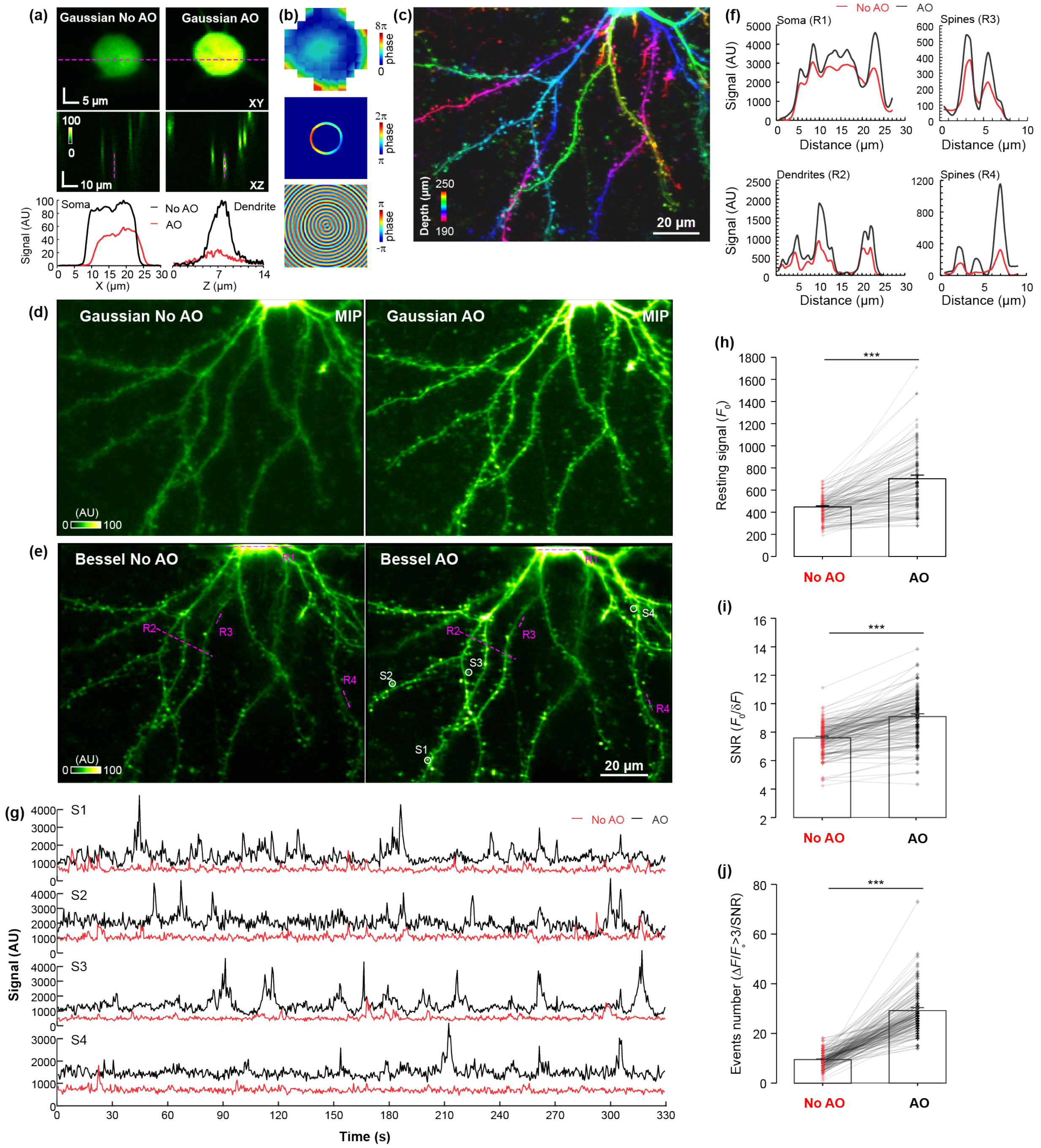

Supplementary Fig. 10. AO enhances detection of spontaneous calcium transients in volumetric imaging of dendritic spines in the awake mouse V1 *in vivo*. Mouse primary visual cortex sparsely expressing the genetically encoded calcium indicator GCaMP6s was imaged through a cranial window *in vivo*. (**a**) (upper) Lateral images of a soma (Z = 220 µm) and (middle) axial images of its nearby dendrites measured without and with AO correction of Gaussian focus; (lower) Signal profiles along the dashed purple lines. Aberration measurement was performed using the soma. (**b**) Wavefront on pupil plane/SLM2 for aberration correction of Gaussian focus, after transmitted through the annular mask, and the computed focal-plane pattern on SLM1 for aberration-corrected Bessel focus. (**c**) Gaussian imaging stack from Z =

190 μm to Z = 250 μm color-coded by depth. (**d**) Mean intensity projections (MIP) of Gaussian imaging stacks measured without and with AO. (**e**) Bessel images of the same volume as in **d**, obtained without and with AO. (**f**) Signal profiles along the dashed purple lines (R1-4) in **e**. (**g**) Four representative calcium traces from spines in white circles (S1-4) in **e**, indicative of spontaneous activity measured without and with AO. (**h-j**) Basal fluorescent signal (F_0), signal-to-noise ratio (SNR, the ratio of F_0 and its standard deviation σ), and number of detected spontaneous calcium events of n=140 spines in the primary visual cortex of a mouse in this volume measured without and with AO, respectively. Box plots: center line, mean; whiskers, +/- SEM. Two-sided paired t-test, ***: $p < 0.001$. Post-objective powers: 48 mW for Gaussian and 97 mW for Bessel. Wavelength: 940 nm. AU: arbitrary unit.

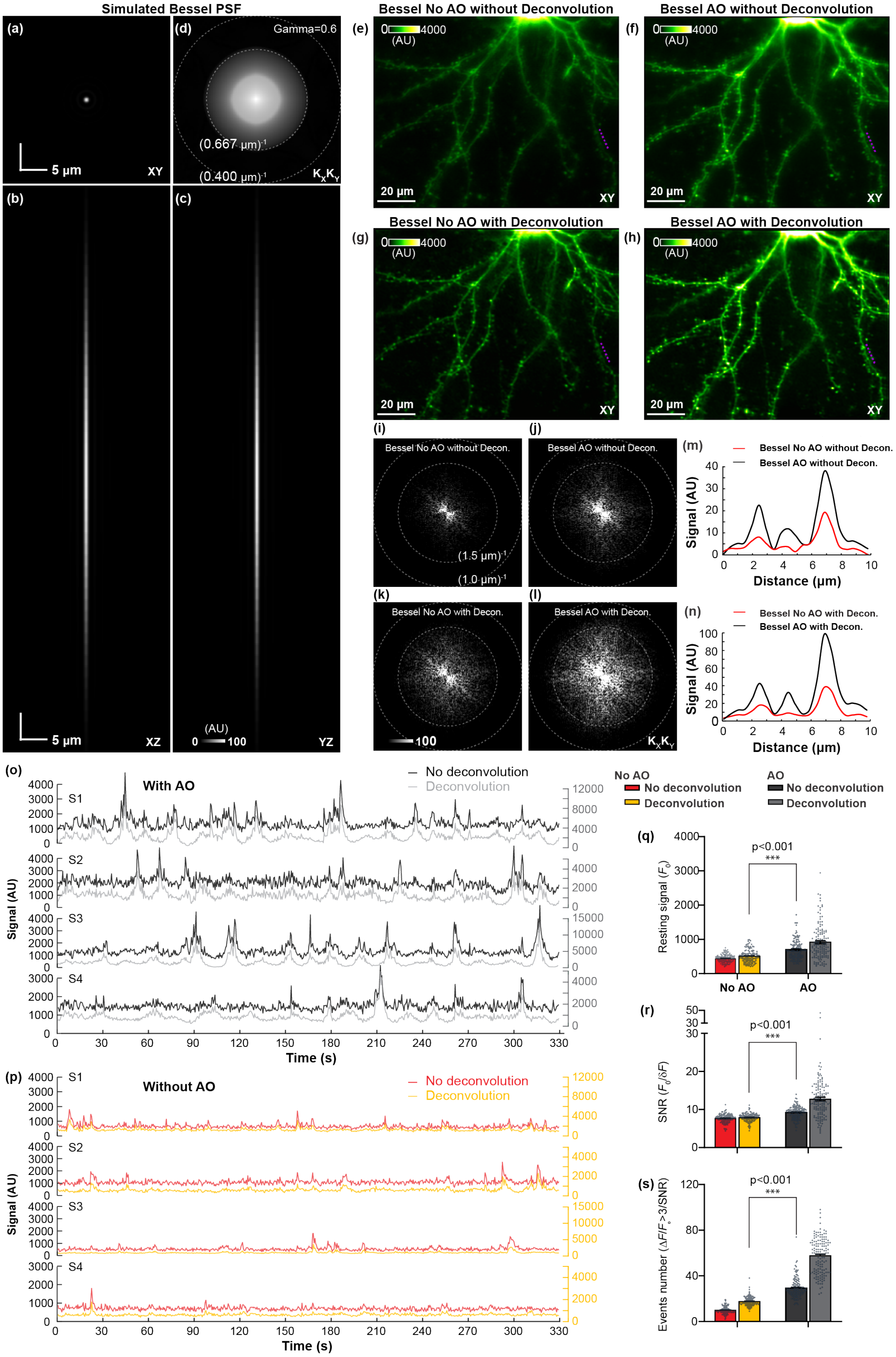

Simulated Bessel PSF
(a)
5 µm
XY
(d)
Gamma=0.6
(0.667 µm)-1
(0.400 µm)-1
KxKy
(b)
5 µm
XZ
(c)
(AU)
0 100
YZ
Bessel No AO without Deconvolution
Bessel AO without Deconvolution
(e)
(f)
0 4000
(AU)
20 µm
XY
Bessel No AO with Deconvolution
Bessel AO with Deconvolution
(g)
(h)
(i)
(j)
Bessel No AO without Decon.
Bessel AO without Decon.
(1.5 µm)-1
(1.0 µm)-1
(k)
(l)
Bessel No AO with Decon.
Bessel AO with Decon.
100
KxKy
(m)
(n)
Signal (AU)
Distance (µm)
(o)
With AO
No deconvolution
Deconvolution
S1
S2
S3
S4
Signal (AU)
Time (s)
(p)
Without AO
No AO
AO
No deconvolution
Deconvolution
(q)
Resting signal (F0)
p<0.001
***
(r)
SNR (F0/δF)
(s)
Events number (ΔF/F0>3/SNR)

Supplementary Fig. 11. Effects of deconvolution on Bessel images measured without and with AO. Demonstrated by dendrites and dendritic spines of a neuron in V1 expressing GCaMP6s and imaged through a cranial window *in vivo* with Bessel focus (**a**) Lateral PSF of the numerically simulated Bessel focus (Z = 0 μm). (**b, c**) Axial PSFs (XZ and YZ) of the simulated Bessel focus (Z = -50 μm to 50 μm). Numerical sampling resolution: $\Delta X = \Delta Y = 0.02$ μm, $\Delta Z = 2$ μm. (**d**) Optical transfer function of the simulated Bessel PSF in the spatial frequency space ($K_X K_Y$). Dashed circle: diffraction limit. (**e, f**) Bessel images without and with AO correction, respectively. (**g, h**) Deconvoluted images from **e** and **f**, respectively, via Richardson–Lucy deconvolution algorithm using the numerically simulated Bessel PSF. (**i**, **j**, **k**, **l**) Spectral power in the spatial frequency space ($K_X K_Y$) for images in **e**, **f**, **g**, **h**, respectively. Dashed circle: diffraction limit. (**m, n**) Signal profiles along the dashed purple lines in **e** and **f**, **g** and **h**, respectively. (**o, p**) Representative calcium traces of the same dendritic spines measured with (**o**) and without (**p**) AO correction, extracted with (grey and yellow curves) and without (black and red curves) deconvolution. (**q-s**) Resting fluorescent signal (**q**), signal-to-noise ratio(**r**), and number of detected spontaneous calcium events (**s**) of n=140 spines in the primary visual cortex of a mouse within the imaged volume, measured before and after the AO correction, analyzed without and with the deconvolution. Box plots: center line, mean; whiskers, +/- SEM. Two-sided t-tests were performed between No AO with deconvolution and AO without deconvolution data. AU: arbitrary unit.

(a)

Bessel module

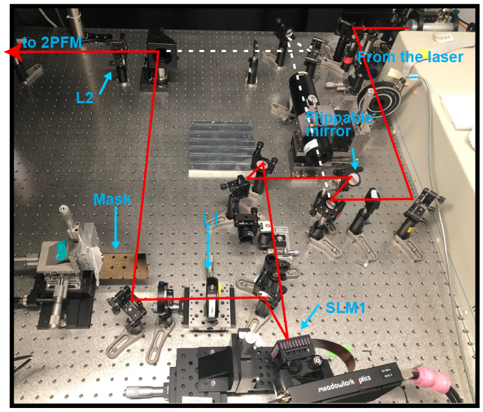

(b)

Part list of the Bessel module

Part	Specification	Manufacturer
Flip mirror mount	Kinematic Flip Base, Threaded 8-32 Mounting	Newport
SLM1 (Focal)	1920 × 1152 Spatial Light Modulator	Meadowlark Optics
L1	f=200 mm	Thorlabs
Mask	Inner diameter =1.015 mm, Outer diameter=1.2 mm	Photo Sciences, Inc.
3D translational stage for Mask	XYZ Linear Stage, ULTRAlign, 25 mm, Crossed-Roller, 8-32 & 1/4-20	Newport
L2	f=750 mm	Thorlabs
L3 (not shown in photo)	f=350 mm	Thorlabs
SLM2 (within 2PFM, not shown in photo)	512 × 512 Spatial Light Modulator	Meadowlark
Two-photon fluorescence microscope (2PFM, not shown in photo)	Described in detail in Ref. 17 in the manuscript	

Supplementary Fig. 12. (**a**) Photo of the Bessel module located between excitation laser and 2PFM. The original Gaussian path (dashed white lines) and Bessel path (red lines) can be switched by a flippable mirror. (**b**) Part list of the Bessel module.

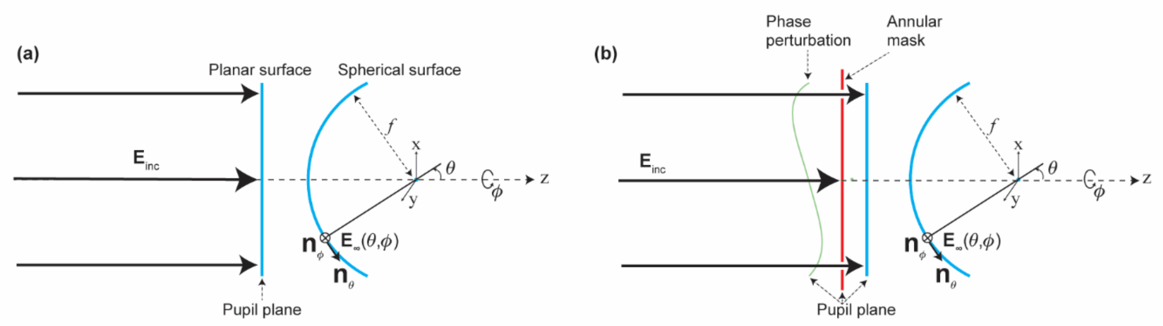

Supplementary Fig. 13. Calculate Bessel PSF from an incident wavefront distorted by noncircularly symmetric aberrations. (**a**) Geometrical representation of the model and definition of coordinates. (**b**) Calculation of aberrated Bessel PSF after applying phase perturbation and annular amplitude mask.

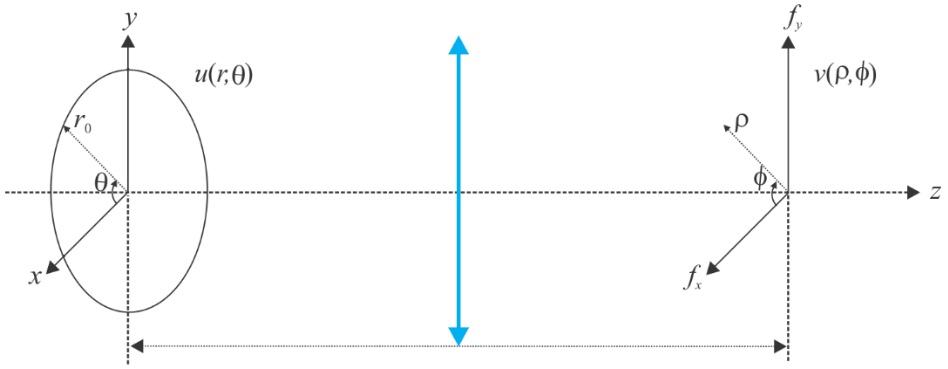

Supplementary Fig. 14. Geometrical representation of the coordinates.

Supplementary Table 1. Imaging settings and sample information for all biological imaging experiments.

Parameters	Fig. 3	Fig. 4	Fig. 5
Sample	In vivo mouse brain (Thy1-GFP-M)	In vivo mouse brain (C57BL/6J, Cre-dependent GCaMP7s)	In vivo mouse brain (C57BL/6J, Cre-dependent iGluSnFR-A184S)
Imaging depth	200-260 µm	340-400 µm	60-180 µm
Gaussian focus post-objective power	36 mW	108 mW	92 mW
Bessel focus post-objective power	78 mW	97 mW	116 mW
FOV & pixel size	Large FOV: 128×128×60 µm^3 @ 0.5µm<br>Small FOV: 51.2×40×60 µm^3 @ 0.2µm	Gaussian: 128×128×60 µm^3 @ 0.5µm<br>Bessel: 128×100×60 µm^3 @ 0.5µm	Gaussian: 128×128×120 µm^3 @ 0.5µm<br>Bessel: 128×100×120 µm^3 @ 0.5µm
Gaussian frame rate	3.3 fps	3.3 fps	3.3 fps
Bessel volume rate	3.3 volume per second	1.5 volume per second	1.5 volume per second
Parameters	Fig. S5	Fig. S6	Fig. S7
Sample	In vivo mouse brain (Thy1-GFP-M)	In vivo mouse brain (Thy1-GFP-M)	In vivo zebrafish larval (isl1:GFP)
Imaging depth	40-100 µm	480-540 µm	80-140 µm
Gaussian focus post-objective power	27 mW	116 mW	30 mW
Bessel focus post-objective power	62 mW	102 mW	90 mW
FOV & pixel size	128×128×60 µm^3 @ 0.5µm	128×128×60 µm^3 @ 0.5µm	128×128×60 µm^3 @ 0.5µm
Gaussian frame rate	3.3 fps	3.3 fps	3.3 fps
Bessel volume rate	1.3 volume per second	1.3 volume per second	3.3 volume per second
Parameters	Fig. S8		
Sample	In vivo mouse brain (C57BL/6J, Cre-dependent GCaMP6s)		
Imaging depth	190-250 µm		
Gaussian focus post-objective power	48 mW		
Bessel focus post-objective power	97 mW		
FOV & pixel size	128×100×60 µm^3 @ 0.5µm		
Gaussian frame rate	3.3 fps		
Bessel volume rate	1.5 volume per second		

Supplementary References

1. J. Ericson, S. Thor, T. Edlund, T. M. Jessell, T. Yamada, Early Stages of Motor Neuron Differentiation Revealed by Expression of Homeobox Gene Islet-1. *Science* **256**, 1555-1560 (1992).
2. S.-i. Higashijima, Y. Hotta, H. Okamoto, Visualization of Cranial Motor Neurons in Live Transgenic Zebrafish Expressing Green Fluorescent Protein Under the Control of the *Islet-1* Promoter/Enhancer. *The Journal of Neuroscience* **20**, 206-218 (2000).
3. K. Wang *et al.*, Rapid adaptive optical recovery of optimal resolution over large volumes. *Nature Methods* **11**, 625 (2014).
4. K. Svoboda, W. Denk, D. Kleinfeld, D. W. Tank, In vivo dendritic calcium dynamics in neocortical pyramidal neurons. *Nature* **385**, 161-165 (1997).
5. R. Yuste, W. Denk, Dendritic spines as basic functional units of neuronal integration. *Nature* **375**, 682-684 (1995).
6. R. Lu *et al.*, Video-rate volumetric functional imaging of the brain at synaptic resolution. *Nature Neuroscience* **20**, 620 (2017).
7. R. Lu *et al.*, Rapid mesoscale volumetric imaging of neural activity with synaptic resolution. *Nature Methods* **17**, 291-294 (2020).
8. R. Lu, M. Tanimoto, M. Koyama, N. Ji, 50 Hz volumetric functional imaging with continuously adjustable depth of focus. *Biomed. Opt. Express* **9**, 1964-1976 (2018).
9. T.-W. Chen *et al.*, Ultrasensitive fluorescent proteins for imaging neuronal activity. *Nature* **499**, 295 (2013).
10. L. Novotny, B. Hecht, *Principles of nano-optics* (Cambridge university press, 2012).
11. M. Born, E. Wolf, *Principles of optics: electromagnetic theory of propagation, interference and diffraction of light* (Elsevier, 2013).
12. R. J. Mathar, Zernike basis to cartesian transformations. *arXiv preprint arXiv:0809.2368* (2008).
