# Supplementary material for: In vivo volumetric imaging of calcium and glutamate activity at synapses with high spatiotemporal resolution
Source: Nat Commun. 2021 Nov 16;12:6630. doi: 10.1038/s41467-021-26965-7 (PMC8595604; doi:10.1038/s41467-021-26965-7)
Supplement: Supplementary file 2 — Reporting Summary [file 41467_2021_26965_MOESM2_ESM.pdf]

nature portfolio

Corresponding author(s): Na Ji

Last updated by author(s): Oct 12, 2021

Reporting Summary

Nature Portfolio wishes to improve the reproducibility of the work that we publish. This form provides structure for consistency and transparency in reporting. For further information on Nature Portfolio policies, see our Editorial Policies and the Editorial Policy Checklist.

Statistics

For all statistical analyses, confirm that the following items are present in the figure legend, table legend, main text, or Methods section.

n/a	Confirmed	
☐	☒	The exact sample size (n) for each experimental group/condition, given as a discrete number and unit of measurement
☐	☒	A statement on whether measurements were taken from distinct samples or whether the same sample was measured repeatedly
☐	☒	The statistical test(s) used AND whether they are one- or two-sided<br>*Only common tests should be described solely by name; describe more complex techniques in the Methods section.*
☒	☐	A description of all covariates tested
☒	☐	A description of any assumptions or corrections, such as tests of normality and adjustment for multiple comparisons
☐	☒	A full description of the statistical parameters including central tendency (e.g. means) or other basic estimates (e.g. regression coefficient) AND variation (e.g. standard deviation) or associated estimates of uncertainty (e.g. confidence intervals)
☐	☒	For null hypothesis testing, the test statistic (e.g. F, t, r) with confidence intervals, effect sizes, degrees of freedom and P value noted<br>*Give P values as exact values whenever suitable.*
☒	☐	For Bayesian analysis, information on the choice of priors and Markov chain Monte Carlo settings
☒	☐	For hierarchical and complex designs, identification of the appropriate level for tests and full reporting of outcomes
☒	☐	Estimates of effect sizes (e.g. Cohen's d, Pearson's r), indicating how they were calculated

Our web collection on statistics for biologists contains articles on many of the points above.

Software and code

Policy information about availability of computer code

Data collection	A custom LabVIEW (2015 SP1) program was used to acquire the two-photon images.
Data analysis	The MATLAB Codes (R2018a) used to generate the AO patterns have been provided as supplementary codes, and can also be found at https://github.com/JiLabUCBerkeley/AOBessel. Imaging data were processed with Fiji (win64). The non-rigid motion correction algorithm (NoRMCorre, Journal of Neuroscience Methods, vol. 291, pp 83-94, 2017) was used to register the Bessel stacks.

For manuscripts utilizing custom algorithms or software that are central to the research but not yet described in published literature, software must be made available to editors and reviewers. We strongly encourage code deposition in a community repository (e.g. GitHub). See the Nature Portfolio guidelines for submitting code & software for further information.

Data

Policy information about availability of data

All manuscripts must include a data availability statement. This statement should provide the following information, where applicable:

- Accession codes, unique identifiers, or web links for publicly available datasets
- A description of any restrictions on data availability
- For clinical datasets or third party data, please ensure that the statement adheres to our policy

The authors declare that data supporting the findings of this study are available within the paper and its supplementary information files. Source data are provided with this paper.

Field-specific reporting

Please select the one below that is the best fit for your research. If you are not sure, read the appropriate sections before making your selection.

☒ Life sciences ☐ Behavioural & social sciences ☐ Ecological, evolutionary & environmental sciences

For a reference copy of the document with all sections, see nature.com/documents/nr-reporting-summary-flat.pdf

Life sciences study design

All studies must disclose on these points even when the disclosure is negative.

Sample size	The technology has been validated with more than 10 mice for the structural and functional imaging. No sample size calculation was performed. But for each experiment with specific aims, the same imaging protocols were repeated with 2 to 10 mice or zebrafishes, which are sufficient for technical demonstration of our method. For statistical analysis, enough samples (> 10 samples) were acquired to ensure sufficient power to test the statistical significance.AO-besse
Data exclusions	No data exclusion was involved.
Replication	Optical system aberration corrections were performed for the Gaussian and Bessel foci before all imaging sessions and the measurements for Fig. 1b and Fig. 1d were repeatedly four times in different imaging sessions. The measurements of aberrated Gaussian and Bessel PSFs with different Zernike modes (Fig. 2b-d) were measured four times within a single imaging session and results reproduced in two imaging sessions. The imaging of Thy1-GFP mice at different depths were repeated at least ten times with eight mice, with representative images shown in Figs. 3d, 3f, and supplementary Figs. 5a, 5c-e, 7c-d, 8a-b. The imaging of zebrafish larval (supplementary Figs. 9d, 9f) were repeated twice. The imaging of calcium signaling in the mouse primary visual cortex (V1) were repeated three times (Figs. 4b, 4d-f) for visual evoked responses with different mice and two times (Figs. 10a, 10d-e, 11a-c, 11e-h) for spontaneous activity with a mouse. The imaging of glutamate signaling (Fig. 5) in V1 were repeated two times with two mice.
Randomization	Proper randomization process was conducted in this study. No AO and AO measurements were interleaved in experiments. Drifting gratings of different angles were delivered in pseudo-random sequence during visual stimulation experiments.
Blinding	Experiment animals and imaging region of interests were randomly chosen without prior bias. Data analysis was performed using the same method for different groups (AO versus No AO) in the same imaging session.

Reporting for specific materials, systems and methods

We require information from authors about some types of materials, experimental systems and methods used in many studies. Here, indicate whether each material, system or method listed is relevant to your study. If you are not sure if a list item applies to your research, read the appropriate section before selecting a response.

Materials & experimental systems

n/a	Involved in the study	
☒	☐	Antibodies
☒	☐	Eukaryotic cell lines
☒	☐	Palaeontology and archaeology
☐	☒	Animals and other organisms
☒	☐	Human research participants
☒	☐	Clinical data
☒	☐	Dual use research of concern

Methods

n/a	Involved in the study	
☒	☐	ChIP-seq
☒	☐	Flow cytometry
☒	☐	MRI-based neuroimaging

Animals and other organisms

Policy information about studies involving animals; ARRIVE guidelines recommended for reporting animal research

Laboratory animals	All animal experiments were conducted according to the National Institutes of Health guidelines for animal research. Procedures and protocols on mice and zebrafish were approved by the Institutional Animal Care and Use Committee at the University of California, Berkeley (AUP-2020-06-13343). Thy1-GFP mice (males, > 2months old) , Wild-type mice (C57BL/6J, females or males, > 2months old) , Transgenic zebrafish Tg(isl1:GFP) at 5 dpf were used in this study. Mice were housed in an animal facility at UC Berkeley campus with 12 light/12 dark cycle, ambient temperature between 20–26 °C and humidity between 40-60%.
Wild animals	The study did not involve wild animals.
Field-collected samples	The study did not involve field-collected samples.
Ethics oversight	All animal experiments were conducted according to the National Institutes of Health guidelines for animal research. Procedures and protocols on mice and zebrafish were approved by the Institutional Animal Care and Use Committee at the University of California,

Berkeley.

Note that full information on the approval of the study protocol must also be provided in the manuscript.
