# Supplementary material for: In vivo volumetric imaging of calcium and glutamate activity at synapses with high spatiotemporal resolution
Source: Nat Commun. 2021 Nov 16;12:6630. doi: 10.1038/s41467-021-26965-7 (PMC8595604; doi:10.1038/s41467-021-26965-7)
Supplement: Supplementary file 3 — Description of Additional Supplementary Files [file 41467_2021_26965_MOESM3_ESM.pdf]

Title: Supplementary Movie 1.
Description: Volumetric imaging of visually evoked calcium signaling of GCaMP7s$^+$ dendrites and dendritic spines in the awake mouse V1 *in vivo* by Bessel focus scanning without and with AO. Imaging volume: 128 × 128 × 60 μm^3 from Z = 340 μm to Z = 400 μm below pia. Visual stimuli: 12 full-field drifting gratings (0° to 330° at 30° increments) in pseudorandom sequences. Ten trials were repeated for each stimulus and the trial-averaged results were presented in the movie.

Title: Supplementary Movie 2.
Description: Imaging of visually evoked calcium signaling of GCaMP7s$^+$ dendrites and dendritic spines in the awake mouse V1 *in vivo* from a single optical section (Z = 350 μm below pia) by Gaussian focus scanning without and with AO. Visual stimuli: 12 full-field drifting gratings (0° to 330° at 30° increments) in pseudorandom sequences. Ten trials were repeated for each stimulus and the trial-averaged results were presented in the movie.

Title: Supplementary Movie 3.
Description: Spontaneous calcium transients of GCaMP6s$^+$ dendrites and dendritic spines in a volume (128 × 100 × 60 μm^3 from Z = 190 μm to Z = 250 μm below pia) imaged by Bessel focus scanning without and with AO at 2 Hz volume rate.

Title: Supplementary Movie 4.
Description: Spontaneous calcium transients of GCaMP6s+ dendrites and dendritic spines at a single optical section (128 × 100 μm^2 at Z = 220 μm below pia) imaged by Gaussian focus scanning without and with AO at 3.3 Hz frame rate.

Title: Supplementary Movie 5.
Description: Volumetric imaging of visually evoked glutamate signaling (iGluSnFR-A184S) in apical dendritic spines of a visual cortical neuron in an awake mouse *in vivo* by Bessel focus scanning without and with AO (128 × 128 × 60 μm^3 from Z = 60 μm to Z = 120 μm below pia). Visual stimuli: 12 full-field drifting gratings (0° to 330° at 30° increments) in pseudorandom sequences. Twenty trials were repeated for each stimulus and the trial-averaged results were presented in the movie.

Title: Supplementary Movie 6.
Description: Volumetric imaging of visually evoked glutamate signaling (iGluSnFR-A184S) in basal dendritic spines of a visual cortical neuron in an awake mouse *in vivo* by Bessel focus scanning without and with AO (128 × 128 × 60 μm^3 from Z = 120 μm to Z = 180 μm below pia). Visual stimuli: 12 full-field drifting gratings (0° to 330° at 30° increments) in pseudorandom sequences. Twenty trials were repeated for each stimulus and the trial-averaged results were presented in the movie.

Title: Supplementary Software.
Description: MATLAB® codes used to generate the AO-corrected Bessel phase patterns and to calculate PSF for foci experiencing non-circularly-symmetric aberrations are included in Supplementary Software.
