# Supplementary material for: In vivo volumetric imaging of calcium and glutamate activity at synapses with high spatiotemporal resolution
Source: Nat Commun. 2021 Nov 16;12:6630. doi: 10.1038/s41467-021-26965-7 (PMC8595604; doi:10.1038/s41467-021-26965-7)
Supplement: Supplementary file 4 — Supplementary Software [file 41467_2021_26965_MOESM4_ESM.zip › Supplementary Codes/Read me.pdf]

The supplementary codes are developed in MATLAB R2018a with Windows 10 (recommend Intel Xeon Processor E5-2667, RAM>32 GB). Related functions have been included in the same folder for each code. The purposes and usages of the supplementary codes are listed below with the code procedures to descript the functionality.

Code 1 - Calculate AO Bessel Phase Mask:

Purpose: This code is used to generate the phase pattern on SLM1/"Bessel SLM" ("AO Bessel phase.tif" and "AO_Bessel_Focal.bmp") using the measured AO corrective wavefront at the objective pupil plane ("AO_pupil.tif"). Applying this phase pattern on SLM1 leads to an aberration-corrected Bessel focus at the objective focal plane.

Usage: Open the "AO_Bessel.m" file in the folder to run the main function. Load the "AO_pupil.tif" file in the same folder when the program requests. Then the AO phase pattern that would generate an aberration-corrected Bessel focus will be saved in the same folder as "AO_Bessel_Focal.bmp" file, which is then displayed on the Bessel SLM. Some intermediate results, including the electric fields generated by the calculated phase pattern at the mask plane, "AO Bessel at Mask", are saved in the same folder. The calculation takes about 5 minutes on a typical computer.

Code procedures:

1. Start the main program "AO_Bessel.m"

Specify the system parameters (SLM pixel size, pixel number, wavelength, magnification, et al)

Specify the transmissive mask parameters (inner diameter, outer diameter), which determines the NA and axial length of a conventional Bessel focus

Calculate the concentric binary phase profile on Bessel SLM that would generate the conventional Bessel focus described above using "AnnularGratingOnSLMGenerator.m"

2. Initialize an electric field (Field_SML1_0) for the Bessel SLM conjugated to the focal plane on Bessel SLM

Assign a Gaussian amplitude profile to the electric field

Assign the concentric binary phase profile calculated in Step 1 to the electric field

3. Fourier transform Field_SML1_0 to the mask conjugated to the pupil plane

Do a one-dimensional Fourier transform on Field_SML1_0 with "Fourier_CircularLens.m"

Display the resulting amplitude and phase profile in Figure 1 (red dashed lines indicate the location of transmissive annulus in the mask)

4. Propagate the measured AO correction phase at the pupil plane to the mask plane

Load AO correction phase measured at the pupil plane ("AO_pupil.tif")

Sample the AO correction phase with 3 µm per pixel (upsampling from the SLM pixel size of 15 µm for numerical calculation)

Propagate an electric field with a Gaussian amplitude profile and the AO correction phase profile to the mask based on the system magnification (Magnification_SLM2toMask)

Rotate the orientation based on how many times the electrical field at the pupil SLM is conjugated to the electrical field at the mask ("ConjugateTimes") (e.g., 180 degree if the ConjugateTimes is odd)

Obtain the required phase profile within the transmissive annulus on the mask

5. Inverse Fourier transform the required AO correction phase profile to the Bessel SLM located at the focal plane

Construct an annular electric field with a constant amplitude profile and a phase profile as calculated in Step 4

Do a two-dimensional Fourier transform of the annular electric field with "Calc_Square_Field_FFT_ZeroPadding.m" with zero padding, which gives rise to the phase pattern to be applied to the Bessel SLM to generate an aberration-corrected Bessel focus

Resize this phase pattern to the dimension and pixel size of the Bessel SLM ("AO-Bessel Phase Pattern")

6. Validate the AO-Bessel phase pattern

Construct the electric field at Bessel SLM with "GenerateVirtualSLMGausianFiled.m"

Assign a Gaussian amplitude profile based on the diameter of the beam incident on the Bessel SLM

Assign the AO-Bessel correction pattern phase pattern from Step 5

Propagate this electric field to the mask via a two-dimensional Fourier transform "Calc_Square_Field_FFT_ZeroPadding.m"

Display and compare the phase profiles between that generated by the AO-Bessel phase pattern (Figure 2) and the required phase profile in 4 (Figure 3).

7. Save the AO correction phase pattern for Bessel validated in 6

Save the phase pattern obtained in Step 5 and validated in Step 6 as a .bmp file to be displayed on Bessel SLM during the experiment

Code 2 - Calculate the Bessel PSF aberrated by astigmatism:

Purpose: This code is used to calculate the Bessel PSF aberrated by astigmatism, as an example to simulate the PSF aberrated by non-circular aberrations characterized in Zernike modes.

Usage: Open the "AstiBesselPSF.m" file in the folder to run the main function. The program will load the aberration pattern “Astigmatism.bmp” saved in the same folder and calculate the PSF from Z=-50 µm to 50 µm. Each frame and the 3D stack of the PSF will be saved in the same folder. It is time consuming (~ several hours) to calculate the entire 3D stack, so an example has been saved in the same folder.

Code procedures:

1. Start the main program “AstiBesselPSF.m”

Specify the system parameters (SLM pixel size, pixel number, wavelength, magnification, et al)

Specify the objective parameters (NA, magnification, focal length, et al)

2. Initialize an electric field at the back pupil plane

Load the aberration mode (astigmatism) as the phase pattern

Assign the aberrated phase to the electric field as the phase profile

Assign the annular illumination to the electric field as the amplitude profile

Conjugate the constructed electical field to the objective back pupil plane

Upsample the optical field with a pixel size of 10 µm

3. Specify the dimensions of the calculated 3D PSF

Specify the lateral dimension (x) of the 3D PSF centered at the objective focus

Specify the lateral dimension (y) of the 3D PSF centered at the objective focus

Specify the axial dimension (z) of the 3D PSF centered at the objective focus

4. Calculate the 3D PSF based on a vector diffraction theory

Pass the electric field at the objective back pupil plane from Step 2 and dimensions of the calculated PSF from Step 3 to the function “Calc_Annular_Field_Integrals.m”

“Calc_Annular_Field_Integrals.m” does the integral for each x and y for a cross-section of the 3D PSF based on the vector diffraction theory described in Supplementary 4

Iterate through all z positions to get the 3D PSF

5. Save the 3D PSF into a .tif stack

Save each cross section of the 3D PSF and a tif stack for the entire 3D PSF

Code 3 - - ZernikeModeDecomposition:

Purpose: This code is used to decompose the measured AO correction pattern at the pupil plane into the first 55 Zernike modes. After removing defocus, tip, and tilt, we reconstruct the AO correction pattern (as the sum of the remaining Zernike modes), which can be used to generate the AO-Bessel Phase Pattern by Code 1.

Usage: Open the " ZernikeModeDecomposition.m " file in the folder to run the main function. Load the "AO_wavefront.tif" file in the same folder as the program requests. Then the result will be saved in the same folder as "AO_wavefront_NoDefocus.tif" file. The calculation takes less than 5 minutes in a typical computer.

Code procedures:

1. Start the main program “ZernikeModeDecomposition.m”

Specify the working folder

Load the measured AO correction pattern (in our manuscript, measured with a pupil segmentation method) from the working folder

2. Decompose the AO correction pattern into Zernike Modes

Specify the number of Zernike Modes

Specify the pupil diameter of the AO correction pattern

Decompose the AO correction pattern using function “ZernikeDecomposition.m”

Obtain the Zernike coefficients for the specified Zernike Modes

3. Reconstruct the AO correction patterns

Reconstruct the AO correction pattern based on the full set of specified Zernike modes

Reconstruct the AO correction pattern without defocus, tip, and tilt modes.

4. Display the reconstructed AO correction patterns

Display the Zernike coefficients for the first 55 Zernike modes

Display the original AO correction pattern and the reconstructed AO correction patterns

5. Save the reconstructed AO correction patterns

Save the reconstructed phase patterns in wave numbers.

Code 4 - - SimulationIntensityVariation:

Purpose: This code is used to simulate the amplitude variation of electric field at the objective pupil plane caused by the aberration (astigmatism) occurring 200 mm away from the pupil plane, as well as the effect of focal AO correction. The Bessel PSFs without the amplitude correction via focal AO and after focal AO correction will be save in the same folder.

Usage: Open the "SimulationIntensityVariation.m" file in the folder to run the main function. The program will load the aberration pattern "AstiAO_Cal.tif" saved in the same folder and calculate the amplitude fields with aberration and after focal AO correction. Because it is time consuming (~ several hours) to calculate the entire 3D stack of the Bessel PSF, an example of the Bessel PSF after focal AO correction has been generated and saved in the same folder. By changing the value of flag_focalAO=0 the code simulates Bessel PSF without the amplitude correction via focal AO . By changing the error mode (flag_addPhaseError=0) in the program, the codes can stimulate Bessel PSF without aberration.

Code procedures:

1. Start the main program "SimulationIntensityVariation.m"

Specify the beam type: Gaussian or Bessel

Specify the aberration mode: No aberration or an aberration introduced at a location away from the pupil plane

Specify the aberration position (e.g., Z=200 mm)

Specify the system parameters (e.g., wavelength, objective parameters, and annular illumination parameters)

2. Initialize an electric field at the pupil plane

Assign the amplitude field to be an annular illumination of value 1

Assign the phase field to be a constant value of -π

Construct the electric field with the above amplitude and phase

3. Propagate the optical field to a distance away from the pupil plane

Upsample the electric field in Step 2 to ensure enough resolution

Propagate the electric field away from the pupil plane with the function "propIR.m" (propIR.m function implements the Fresnel Impulse Response Propagator, Z=200 mm)

Update the electric field at the specified distance away from the pupil plane

4. Add phase error at the distance away from the pupil plane

Load an aberration pattern (astigmatism, "AstiAO_Cal.tif" saved in the folder) as the phase error

Add phase error to the phase profile of the electric field calculated in Step 3

5. Propagate the aberrated optical field back to the pupil plane

Propagate the electric field in Step 4 back to the pupil plane with the function "propIR.m", specifying negative distance (e.g., Z=-200 mm)

Display the aberrated electric field (amplitude and phase) at the pupil plane.

Display the focal corrected electric amplitude calculated from Code 1 at the pupil plane.

6. Construct the focal AO corrected Bessel PSF at the pupil plane

Multiple the amplitudes of the aberrated electric field and the focal corrected electric field to construct the corrected amplitude field.

Assign the phase to be a flat phase to construct the corrected electric field

Construct the complex electric field with the above amplitude and phase, in order to simulate the focal AO corrected electric field at the objective back pupil plane

7. Specify the dimensions of the calculated 3D PSF

Specify the lateral dimension (x) of the 3D PSF centered at the objective focus

Specify the lateral dimension (y) of the 3D PSF centered at the objective focus

Specify the axial dimension (z) of the 3D PSF centered at the objective focus

8. Calculate the 3D PSF based on a vector diffraction theory

Pass the optical field at the objective back pupil plane from Step 6 and dimensions of the calculated PSF from Step 7 to the function "Calc_Annular_Field_IntegralsV2.m"

"Calc_Annular_Field_IntegralsV2.m" does the integral for each x and y for a cross-section of the 3D PSF

Iterate through all z positions to get the 3D PSF

9. Save the 3D PSF into a .tif stack

Save each cross section of the 3D PSF and a tif stack for the entire 3D PSF
