# Supplementary figures and images for: In vivo volumetric imaging of calcium and glutamate activity at synapses with high spatiotemporal resolution
Source: Nat Commun. 2021 Nov 16;12:6630. doi: 10.1038/s41467-021-26965-7 (PMC8595604; doi:10.1038/s41467-021-26965-7)

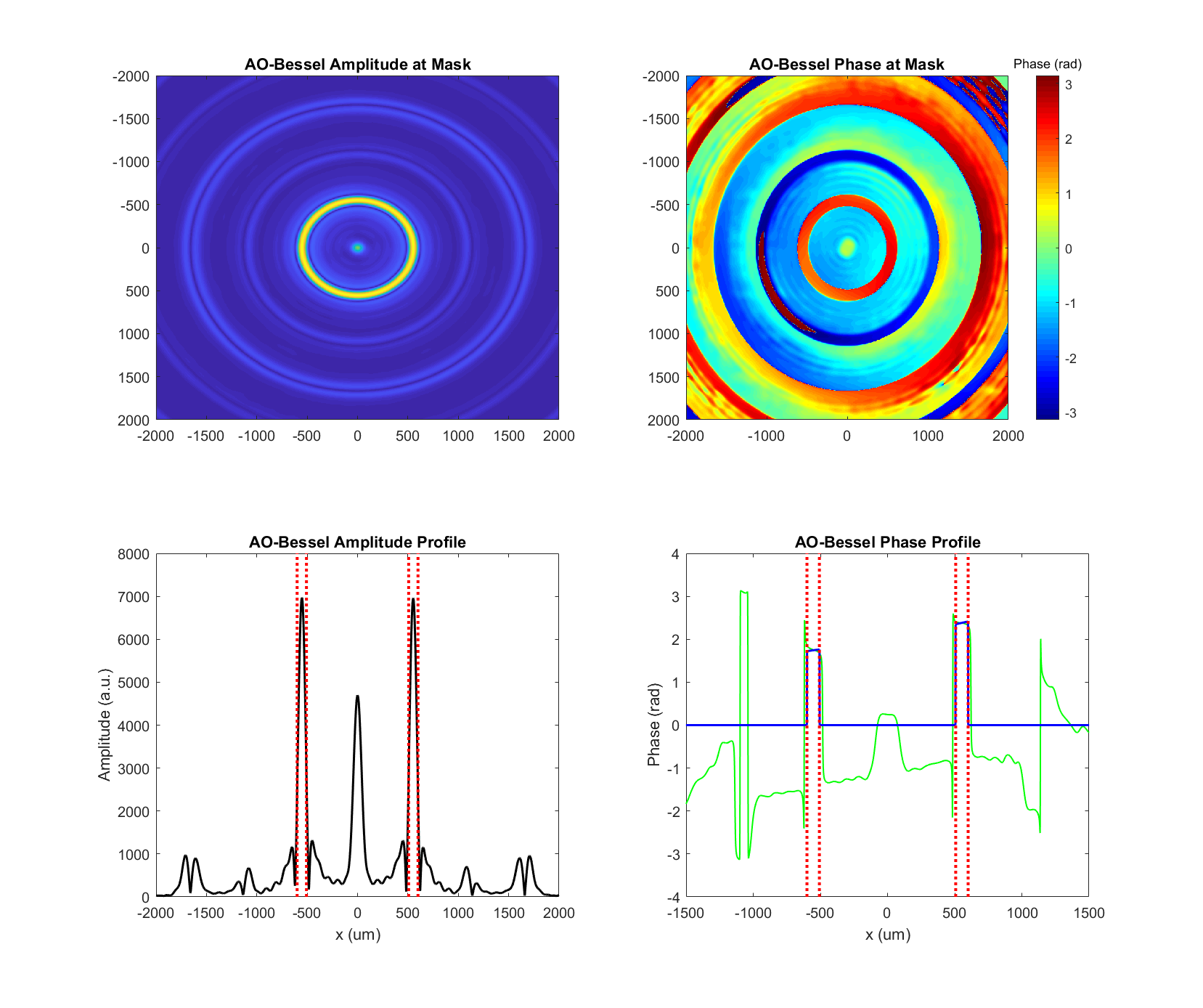

Supplement: Supplementary file 4 — Supplementary Software [file 41467_2021_26965_MOESM4_ESM.zip › Supplementary Codes/Code 1 - Calculate AO Bessel Phase Mask/AO Bessel at Mask.tif]

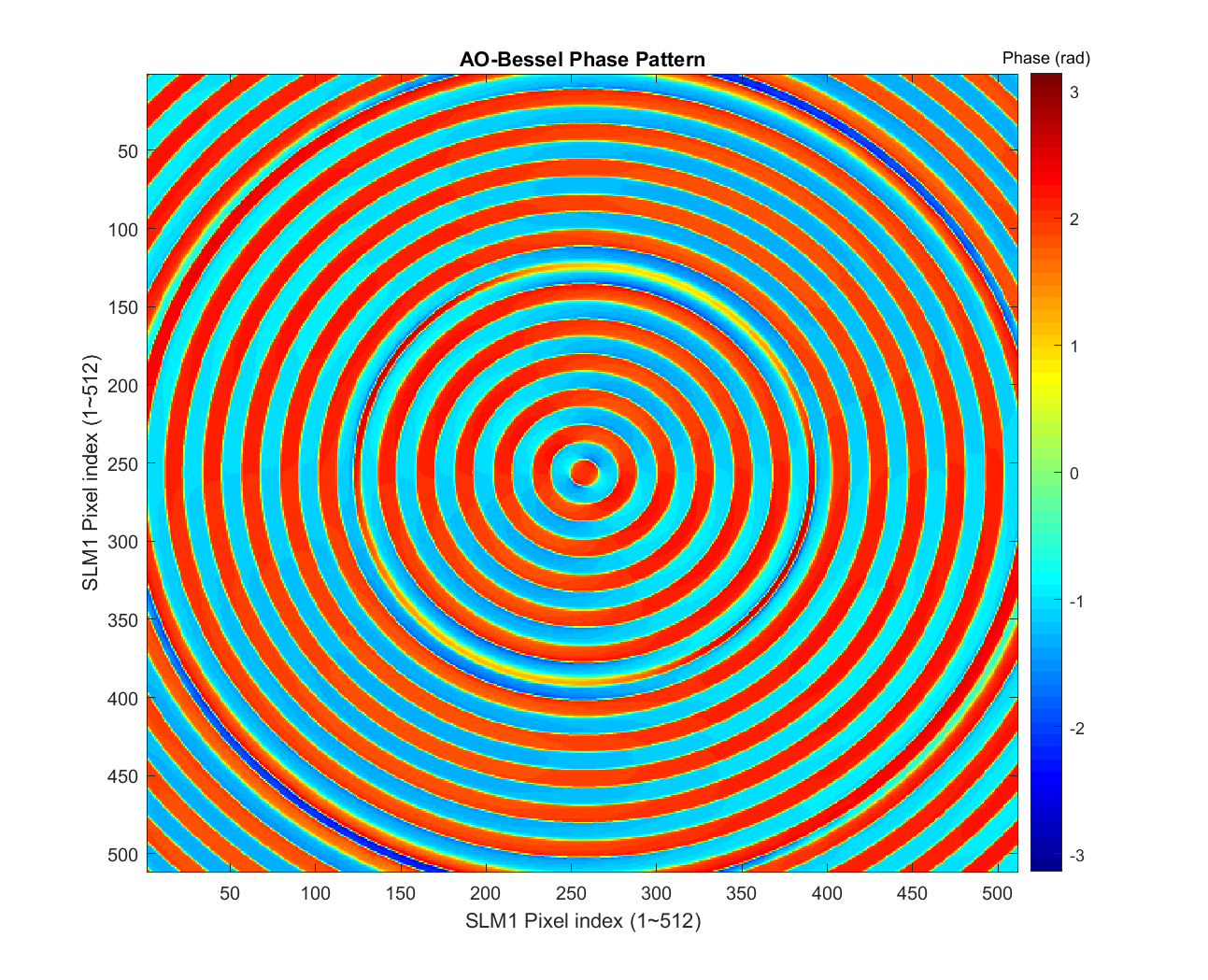

Supplement: Supplementary file 4 — Supplementary Software [file 41467_2021_26965_MOESM4_ESM.zip › Supplementary Codes/Code 1 - Calculate AO Bessel Phase Mask/AO Bessel Phase.tif]

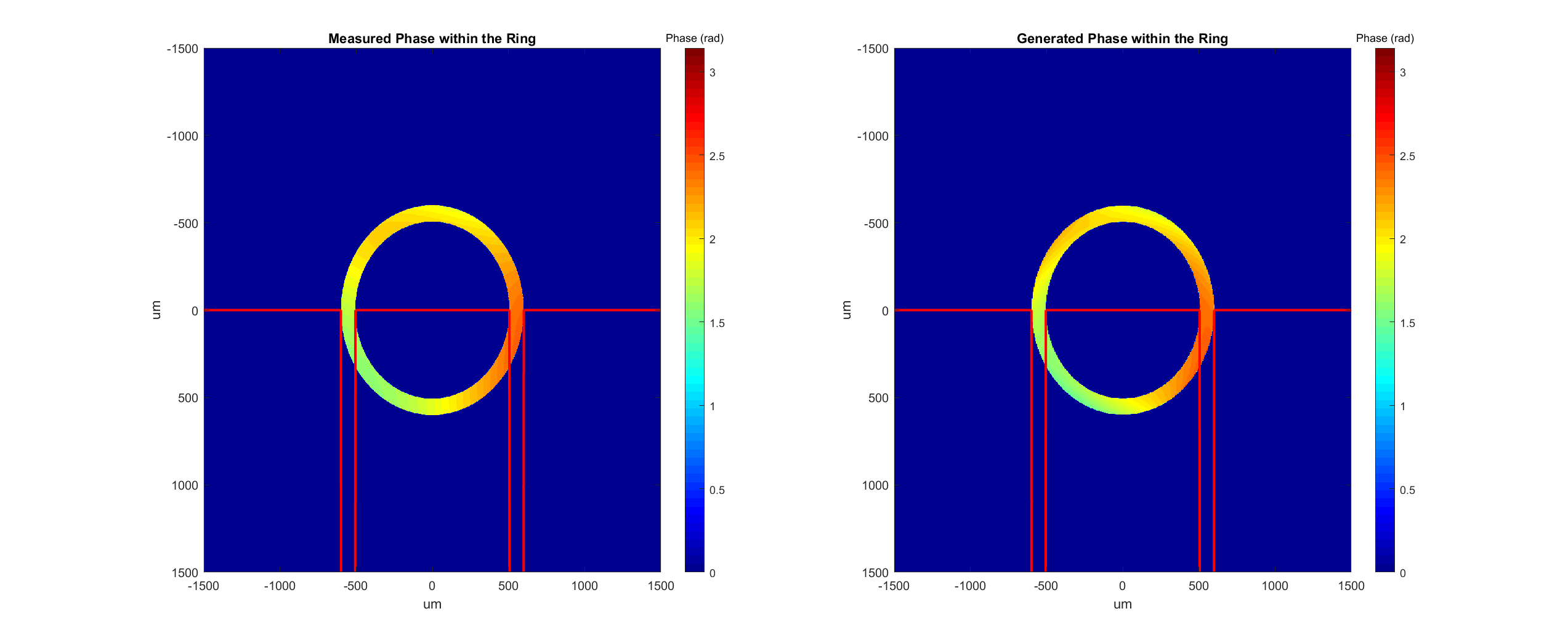

Supplement: Supplementary file 4 — Supplementary Software [file 41467_2021_26965_MOESM4_ESM.zip › Supplementary Codes/Code 1 - Calculate AO Bessel Phase Mask/AO Phase Validate.tif]

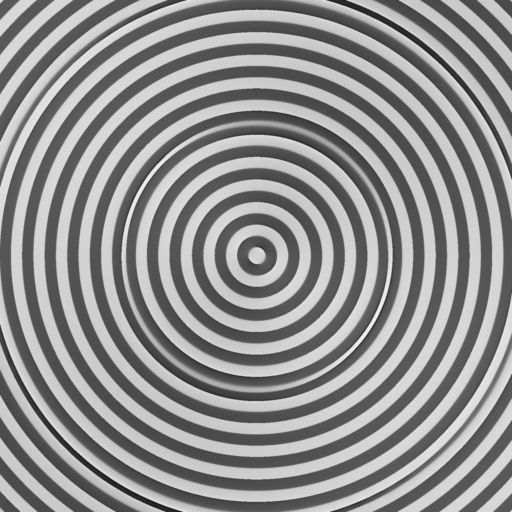

Supplement: Supplementary file 4 — Supplementary Software [file 41467_2021_26965_MOESM4_ESM.zip › Supplementary Codes/Code 1 - Calculate AO Bessel Phase Mask/AO_Bessel_Focal.bmp]

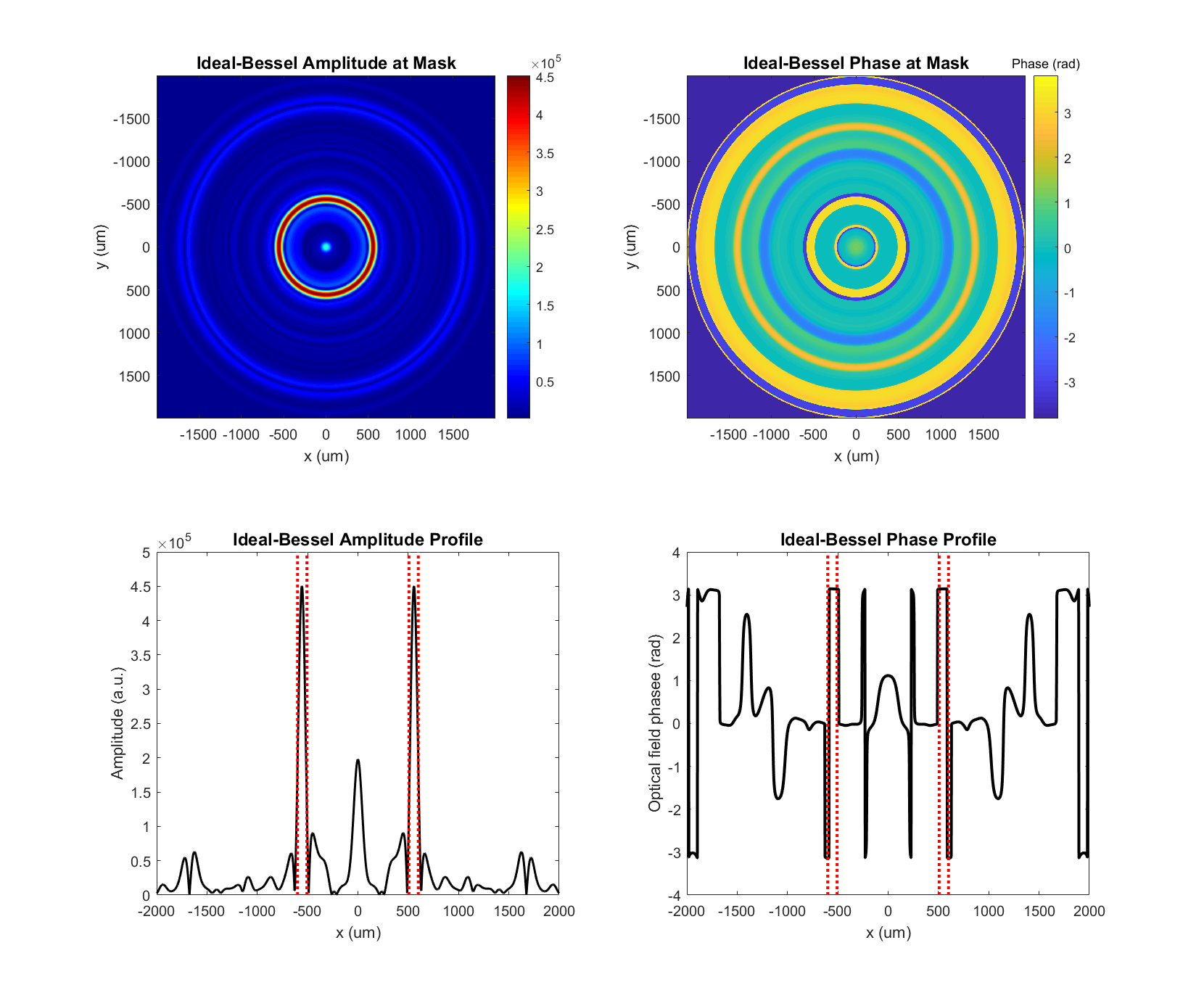

Supplement: Supplementary file 4 — Supplementary Software [file 41467_2021_26965_MOESM4_ESM.zip › Supplementary Codes/Code 1 - Calculate AO Bessel Phase Mask/Ideal Bessel at Mask.tif]

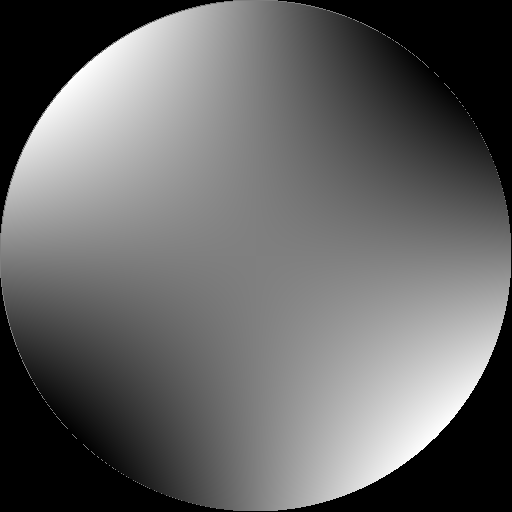

Supplement: Supplementary file 4 — Supplementary Software [file 41467_2021_26965_MOESM4_ESM.zip › Supplementary Codes/Code 2 - Calculate Bessel PSF Aberrated by Astigmatism/Astigmatism.bmp]

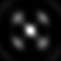

Supplement: Supplementary file 4 — Supplementary Software [file 41467_2021_26965_MOESM4_ESM.zip › Supplementary Codes/Code 2 - Calculate Bessel PSF Aberrated by Astigmatism/asti_3D_0.05xy_1z/-10.tif]

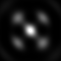

Supplement: Supplementary file 4 — Supplementary Software [file 41467_2021_26965_MOESM4_ESM.zip › Supplementary Codes/Code 2 - Calculate Bessel PSF Aberrated by Astigmatism/asti_3D_0.05xy_1z/-12.tif]

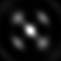

Supplement: Supplementary file 4 — Supplementary Software [file 41467_2021_26965_MOESM4_ESM.zip › Supplementary Codes/Code 2 - Calculate Bessel PSF Aberrated by Astigmatism/asti_3D_0.05xy_1z/-14.tif]

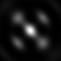

Supplement: Supplementary file 4 — Supplementary Software [file 41467_2021_26965_MOESM4_ESM.zip › Supplementary Codes/Code 2 - Calculate Bessel PSF Aberrated by Astigmatism/asti_3D_0.05xy_1z/-16.tif]

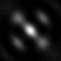

Supplement: Supplementary file 4 — Supplementary Software [file 41467_2021_26965_MOESM4_ESM.zip › Supplementary Codes/Code 2 - Calculate Bessel PSF Aberrated by Astigmatism/asti_3D_0.05xy_1z/-18.tif]

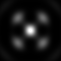

Supplement: Supplementary file 4 — Supplementary Software [file 41467_2021_26965_MOESM4_ESM.zip › Supplementary Codes/Code 2 - Calculate Bessel PSF Aberrated by Astigmatism/asti_3D_0.05xy_1z/-2.tif]

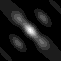

Supplement: Supplementary file 4 — Supplementary Software [file 41467_2021_26965_MOESM4_ESM.zip › Supplementary Codes/Code 2 - Calculate Bessel PSF Aberrated by Astigmatism/asti_3D_0.05xy_1z/-20.tif]

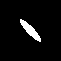

Supplement: Supplementary file 4 — Supplementary Software [file 41467_2021_26965_MOESM4_ESM.zip › Supplementary Codes/Code 2 - Calculate Bessel PSF Aberrated by Astigmatism/asti_3D_0.05xy_1z/-22.tif]

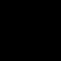

Supplement: Supplementary file 4 — Supplementary Software [file 41467_2021_26965_MOESM4_ESM.zip › Supplementary Codes/Code 2 - Calculate Bessel PSF Aberrated by Astigmatism/asti_3D_0.05xy_1z/-24.tif]

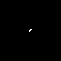

Supplement: Supplementary file 4 — Supplementary Software [file 41467_2021_26965_MOESM4_ESM.zip › Supplementary Codes/Code 2 - Calculate Bessel PSF Aberrated by Astigmatism/asti_3D_0.05xy_1z/-28.tif]

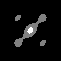

Supplement: Supplementary file 4 — Supplementary Software [file 41467_2021_26965_MOESM4_ESM.zip › Supplementary Codes/Code 2 - Calculate Bessel PSF Aberrated by Astigmatism/asti_3D_0.05xy_1z/-30.tif]

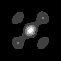

Supplement: Supplementary file 4 — Supplementary Software [file 41467_2021_26965_MOESM4_ESM.zip › Supplementary Codes/Code 2 - Calculate Bessel PSF Aberrated by Astigmatism/asti_3D_0.05xy_1z/-32.tif]

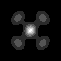

Supplement: Supplementary file 4 — Supplementary Software [file 41467_2021_26965_MOESM4_ESM.zip › Supplementary Codes/Code 2 - Calculate Bessel PSF Aberrated by Astigmatism/asti_3D_0.05xy_1z/-34.tif]

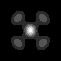

Supplement: Supplementary file 4 — Supplementary Software [file 41467_2021_26965_MOESM4_ESM.zip › Supplementary Codes/Code 2 - Calculate Bessel PSF Aberrated by Astigmatism/asti_3D_0.05xy_1z/-36.tif]

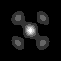

Supplement: Supplementary file 4 — Supplementary Software [file 41467_2021_26965_MOESM4_ESM.zip › Supplementary Codes/Code 2 - Calculate Bessel PSF Aberrated by Astigmatism/asti_3D_0.05xy_1z/-38.tif]

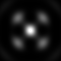

Supplement: Supplementary file 4 — Supplementary Software [file 41467_2021_26965_MOESM4_ESM.zip › Supplementary Codes/Code 2 - Calculate Bessel PSF Aberrated by Astigmatism/asti_3D_0.05xy_1z/-4.tif]

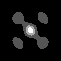

Supplement: Supplementary file 4 — Supplementary Software [file 41467_2021_26965_MOESM4_ESM.zip › Supplementary Codes/Code 2 - Calculate Bessel PSF Aberrated by Astigmatism/asti_3D_0.05xy_1z/-40.tif]

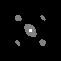

Supplement: Supplementary file 4 — Supplementary Software [file 41467_2021_26965_MOESM4_ESM.zip › Supplementary Codes/Code 2 - Calculate Bessel PSF Aberrated by Astigmatism/asti_3D_0.05xy_1z/-42.tif]

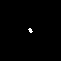

Supplement: Supplementary file 4 — Supplementary Software [file 41467_2021_26965_MOESM4_ESM.zip › Supplementary Codes/Code 2 - Calculate Bessel PSF Aberrated by Astigmatism/asti_3D_0.05xy_1z/-44.tif]

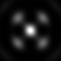

Supplement: Supplementary file 4 — Supplementary Software [file 41467_2021_26965_MOESM4_ESM.zip › Supplementary Codes/Code 2 - Calculate Bessel PSF Aberrated by Astigmatism/asti_3D_0.05xy_1z/-6.tif]

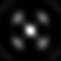

Supplement: Supplementary file 4 — Supplementary Software [file 41467_2021_26965_MOESM4_ESM.zip › Supplementary Codes/Code 2 - Calculate Bessel PSF Aberrated by Astigmatism/asti_3D_0.05xy_1z/-8.tif]

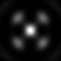

Supplement: Supplementary file 4 — Supplementary Software [file 41467_2021_26965_MOESM4_ESM.zip › Supplementary Codes/Code 2 - Calculate Bessel PSF Aberrated by Astigmatism/asti_3D_0.05xy_1z/0.tif]

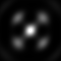

Supplement: Supplementary file 4 — Supplementary Software [file 41467_2021_26965_MOESM4_ESM.zip › Supplementary Codes/Code 2 - Calculate Bessel PSF Aberrated by Astigmatism/asti_3D_0.05xy_1z/10.tif]

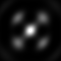

Supplement: Supplementary file 4 — Supplementary Software [file 41467_2021_26965_MOESM4_ESM.zip › Supplementary Codes/Code 2 - Calculate Bessel PSF Aberrated by Astigmatism/asti_3D_0.05xy_1z/12.tif]

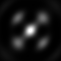

Supplement: Supplementary file 4 — Supplementary Software [file 41467_2021_26965_MOESM4_ESM.zip › Supplementary Codes/Code 2 - Calculate Bessel PSF Aberrated by Astigmatism/asti_3D_0.05xy_1z/14.tif]

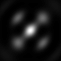

Supplement: Supplementary file 4 — Supplementary Software [file 41467_2021_26965_MOESM4_ESM.zip › Supplementary Codes/Code 2 - Calculate Bessel PSF Aberrated by Astigmatism/asti_3D_0.05xy_1z/16.tif]

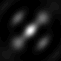

Supplement: Supplementary file 4 — Supplementary Software [file 41467_2021_26965_MOESM4_ESM.zip › Supplementary Codes/Code 2 - Calculate Bessel PSF Aberrated by Astigmatism/asti_3D_0.05xy_1z/18.tif]

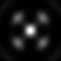

Supplement: Supplementary file 4 — Supplementary Software [file 41467_2021_26965_MOESM4_ESM.zip › Supplementary Codes/Code 2 - Calculate Bessel PSF Aberrated by Astigmatism/asti_3D_0.05xy_1z/2.tif]

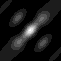

Supplement: Supplementary file 4 — Supplementary Software [file 41467_2021_26965_MOESM4_ESM.zip › Supplementary Codes/Code 2 - Calculate Bessel PSF Aberrated by Astigmatism/asti_3D_0.05xy_1z/20.tif]

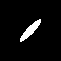

Supplement: Supplementary file 4 — Supplementary Software [file 41467_2021_26965_MOESM4_ESM.zip › Supplementary Codes/Code 2 - Calculate Bessel PSF Aberrated by Astigmatism/asti_3D_0.05xy_1z/22.tif]

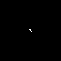

Supplement: Supplementary file 4 — Supplementary Software [file 41467_2021_26965_MOESM4_ESM.zip › Supplementary Codes/Code 2 - Calculate Bessel PSF Aberrated by Astigmatism/asti_3D_0.05xy_1z/28.tif]

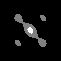

Supplement: Supplementary file 4 — Supplementary Software [file 41467_2021_26965_MOESM4_ESM.zip › Supplementary Codes/Code 2 - Calculate Bessel PSF Aberrated by Astigmatism/asti_3D_0.05xy_1z/30.tif]

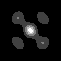

Supplement: Supplementary file 4 — Supplementary Software [file 41467_2021_26965_MOESM4_ESM.zip › Supplementary Codes/Code 2 - Calculate Bessel PSF Aberrated by Astigmatism/asti_3D_0.05xy_1z/32.tif]

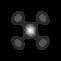

Supplement: Supplementary file 4 — Supplementary Software [file 41467_2021_26965_MOESM4_ESM.zip › Supplementary Codes/Code 2 - Calculate Bessel PSF Aberrated by Astigmatism/asti_3D_0.05xy_1z/34.tif]

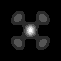

Supplement: Supplementary file 4 — Supplementary Software [file 41467_2021_26965_MOESM4_ESM.zip › Supplementary Codes/Code 2 - Calculate Bessel PSF Aberrated by Astigmatism/asti_3D_0.05xy_1z/36.tif]

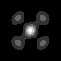

Supplement: Supplementary file 4 — Supplementary Software [file 41467_2021_26965_MOESM4_ESM.zip › Supplementary Codes/Code 2 - Calculate Bessel PSF Aberrated by Astigmatism/asti_3D_0.05xy_1z/38.tif]

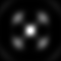

Supplement: Supplementary file 4 — Supplementary Software [file 41467_2021_26965_MOESM4_ESM.zip › Supplementary Codes/Code 2 - Calculate Bessel PSF Aberrated by Astigmatism/asti_3D_0.05xy_1z/4.tif]

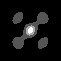

Supplement: Supplementary file 4 — Supplementary Software [file 41467_2021_26965_MOESM4_ESM.zip › Supplementary Codes/Code 2 - Calculate Bessel PSF Aberrated by Astigmatism/asti_3D_0.05xy_1z/40.tif]

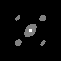

Supplement: Supplementary file 4 — Supplementary Software [file 41467_2021_26965_MOESM4_ESM.zip › Supplementary Codes/Code 2 - Calculate Bessel PSF Aberrated by Astigmatism/asti_3D_0.05xy_1z/42.tif]

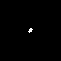

Supplement: Supplementary file 4 — Supplementary Software [file 41467_2021_26965_MOESM4_ESM.zip › Supplementary Codes/Code 2 - Calculate Bessel PSF Aberrated by Astigmatism/asti_3D_0.05xy_1z/44.tif]

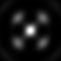

Supplement: Supplementary file 4 — Supplementary Software [file 41467_2021_26965_MOESM4_ESM.zip › Supplementary Codes/Code 2 - Calculate Bessel PSF Aberrated by Astigmatism/asti_3D_0.05xy_1z/6.tif]

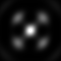

Supplement: Supplementary file 4 — Supplementary Software [file 41467_2021_26965_MOESM4_ESM.zip › Supplementary Codes/Code 2 - Calculate Bessel PSF Aberrated by Astigmatism/asti_3D_0.05xy_1z/8.tif]

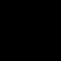

Supplement: Supplementary file 4 — Supplementary Software [file 41467_2021_26965_MOESM4_ESM.zip › Supplementary Codes/Code 2 - Calculate Bessel PSF Aberrated by Astigmatism/asti_3D_0.05xy_1z/asti_stack_0.1xy_1z.tif]

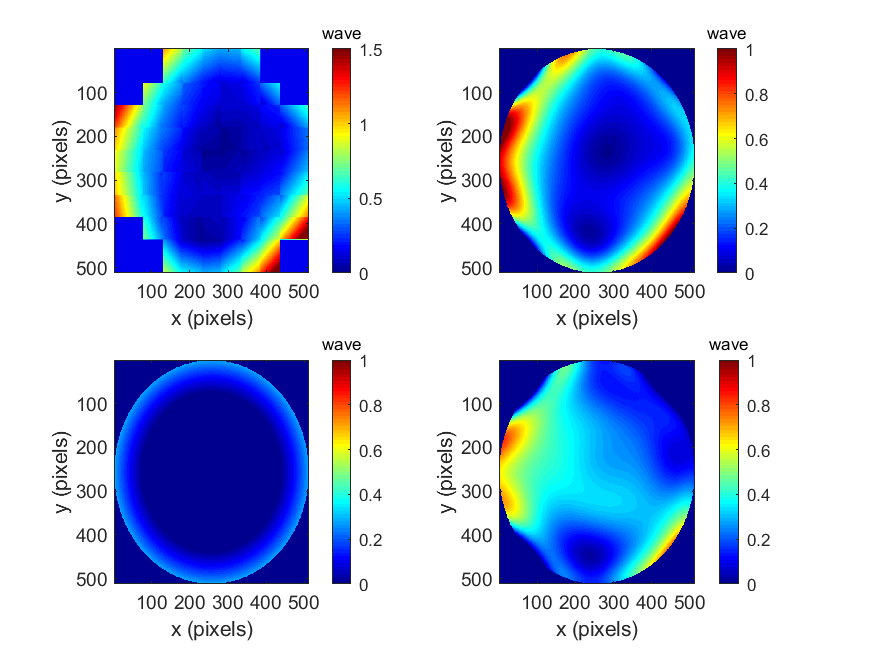

Supplement: Supplementary file 4 — Supplementary Software [file 41467_2021_26965_MOESM4_ESM.zip › Supplementary Codes/Code 3 - ZernikeModeDecomposition/AO_wavefront_ZernikeImg.png]

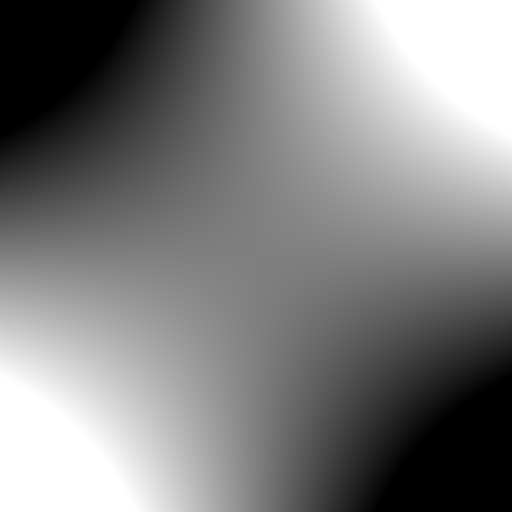

Supplement: Supplementary file 4 — Supplementary Software [file 41467_2021_26965_MOESM4_ESM.zip › Supplementary Codes/Code 4 - SimulationIntensityVariation/AstiAO_Cal.tif]

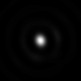

Supplement: Supplementary file 4 — Supplementary Software [file 41467_2021_26965_MOESM4_ESM.zip › Supplementary Codes/Code 4 - SimulationIntensityVariation/FocalAO/-10.tif]

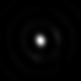

Supplement: Supplementary file 4 — Supplementary Software [file 41467_2021_26965_MOESM4_ESM.zip › Supplementary Codes/Code 4 - SimulationIntensityVariation/FocalAO/-15.tif]

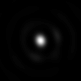

Supplement: Supplementary file 4 — Supplementary Software [file 41467_2021_26965_MOESM4_ESM.zip › Supplementary Codes/Code 4 - SimulationIntensityVariation/FocalAO/-20.tif]

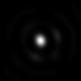

Supplement: Supplementary file 4 — Supplementary Software [file 41467_2021_26965_MOESM4_ESM.zip › Supplementary Codes/Code 4 - SimulationIntensityVariation/FocalAO/-25.tif]

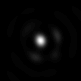

Supplement: Supplementary file 4 — Supplementary Software [file 41467_2021_26965_MOESM4_ESM.zip › Supplementary Codes/Code 4 - SimulationIntensityVariation/FocalAO/-30.tif]

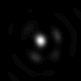

Supplement: Supplementary file 4 — Supplementary Software [file 41467_2021_26965_MOESM4_ESM.zip › Supplementary Codes/Code 4 - SimulationIntensityVariation/FocalAO/-35.tif]

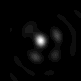

Supplement: Supplementary file 4 — Supplementary Software [file 41467_2021_26965_MOESM4_ESM.zip › Supplementary Codes/Code 4 - SimulationIntensityVariation/FocalAO/-40.tif]

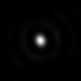

Supplement: Supplementary file 4 — Supplementary Software [file 41467_2021_26965_MOESM4_ESM.zip › Supplementary Codes/Code 4 - SimulationIntensityVariation/FocalAO/-5.tif]

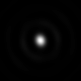

Supplement: Supplementary file 4 — Supplementary Software [file 41467_2021_26965_MOESM4_ESM.zip › Supplementary Codes/Code 4 - SimulationIntensityVariation/FocalAO/0.tif]

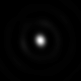

Supplement: Supplementary file 4 — Supplementary Software [file 41467_2021_26965_MOESM4_ESM.zip › Supplementary Codes/Code 4 - SimulationIntensityVariation/FocalAO/10.tif]

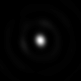

Supplement: Supplementary file 4 — Supplementary Software [file 41467_2021_26965_MOESM4_ESM.zip › Supplementary Codes/Code 4 - SimulationIntensityVariation/FocalAO/15.tif]

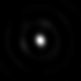

Supplement: Supplementary file 4 — Supplementary Software [file 41467_2021_26965_MOESM4_ESM.zip › Supplementary Codes/Code 4 - SimulationIntensityVariation/FocalAO/20.tif]

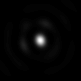

Supplement: Supplementary file 4 — Supplementary Software [file 41467_2021_26965_MOESM4_ESM.zip › Supplementary Codes/Code 4 - SimulationIntensityVariation/FocalAO/25.tif]

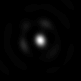

Supplement: Supplementary file 4 — Supplementary Software [file 41467_2021_26965_MOESM4_ESM.zip › Supplementary Codes/Code 4 - SimulationIntensityVariation/FocalAO/30.tif]

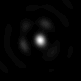

Supplement: Supplementary file 4 — Supplementary Software [file 41467_2021_26965_MOESM4_ESM.zip › Supplementary Codes/Code 4 - SimulationIntensityVariation/FocalAO/35.tif]

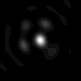

Supplement: Supplementary file 4 — Supplementary Software [file 41467_2021_26965_MOESM4_ESM.zip › Supplementary Codes/Code 4 - SimulationIntensityVariation/FocalAO/40.tif]

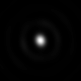

Supplement: Supplementary file 4 — Supplementary Software [file 41467_2021_26965_MOESM4_ESM.zip › Supplementary Codes/Code 4 - SimulationIntensityVariation/FocalAO/5.tif]

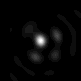

Supplement: Supplementary file 4 — Supplementary Software [file 41467_2021_26965_MOESM4_ESM.zip › Supplementary Codes/Code 4 - SimulationIntensityVariation/FocalAO/Stack_0.05xy_5z.tif]
